# Chemotherapy-induced transposable elements activate MDA5 to enhance haematopoietic regeneration

**DOI:** 10.1038/s41556-021-00707-9

**Published:** 2021-07-12

**Authors:** Thomas Clapes, Aikaterini Polyzou, Pia Prater, Sagar, Antonio Morales-Hernández, Mariana Galvao Ferrarini, Natalie Kehrer, Stylianos Lefkopoulos, Veronica Bergo, Barbara Hummel, Nadine Obier, Daniel Maticzka, Anne Bridgeman, Josip S. Herman, Ibrahim Ilik, Lhéanna Klaeylé, Jan Rehwinkel, Shannon McKinney-Freeman, Rolf Backofen, Asifa Akhtar, Nina Cabezas-Wallscheid, Ritwick Sawarkar, Rita Rebollo, Dominic Grün, Eirini Trompouki

**Affiliations:** 1 Department of Cellular and Molecular Immunology, Max Planck Institute of Immunobiology and Epigenetics, Freiburg, Germany; 2 Faculty of Biology, University of Freiburg, Freiburg, Germany; 3 International Max Planck Research School for Molecular and Cellular Biology (IMPRS-MCB), Freiburg, Germany; 4 Max Planck Institute of Immunobiology and Epigenetics, Freiburg, Germany; 5 Department of Medicine II, Gastroenterology, Hepatology, Endocrinology and Infectious Diseases, Freiburg University Medical Center, Faculty of Medicine, University of Freiburg, Freiburg, Germany; 6 Department of Hematology, St Jude Children’s Research Hospital, Memphis, TN USA; 7 Univ Lyon, INSA-Lyon, INRAE, BF2I, UMR0203, Villeurbanne, France; 8 Department of Computer Science, University of Freiburg, Freiburg, Germany; 9 Medical Research Council Human Immunology Unit, Medical Research Council Weatherall Institute of Molecular Medicine, Radcliffe Department of Medicine, University of Oxford, Oxford, UK; 10 Würzburg Institute of Systems Immunology, Julius-Maximilians-Universität Würzburg, Würzburg, Germany; 11 Department of Chromatin Regulation, Max Planck Institute of Immunobiology and Epigenetics, Freiburg, Germany; 12 Centre for Integrative Biological Signalling Studies (CIBSS), University of Freiburg, Freiburg, Germany; 13 Medical Research Council (MRC), University of Cambridge, Cambridge, UK

**Keywords:** Regeneration, Haematopoiesis, Haematopoietic stem cells, Quiescence

## Abstract

Haematopoietic stem cells (HSCs) are normally quiescent, but have evolved mechanisms to respond to stress. Here, we evaluate haematopoietic regeneration induced by chemotherapy. We detect robust chromatin reorganization followed by increased transcription of transposable elements (TEs) during early recovery. TE transcripts bind to and activate the innate immune receptor melanoma differentiation-associated protein 5 (MDA5) that generates an inflammatory response that is necessary for HSCs to exit quiescence. HSCs that lack MDA5 exhibit an impaired inflammatory response after chemotherapy and retain their quiescence, with consequent better long-term repopulation capacity. We show that the overexpression of ERV and LINE superfamily TE copies in wild-type HSCs, but not in *Mda5*^−/−^ HSCs, results in their cycling. By contrast, after knockdown of LINE1 family copies, HSCs retain their quiescence. Our results show that TE transcripts act as ligands that activate MDA5 during haematopoietic regeneration, thereby enabling HSCs to mount an inflammatory response necessary for their exit from quiescence.

## Abstract (web summary)

Clapes et al. show that chemotherapy leads to chromatin reorganization and increased expression of transposable elements, which promote an MDA5-driven inflammatory response that enhances haematopoietic regeneration.

## Main

HSCs are quiescent under homeostatic conditions to retain their key functional features and maintain a stable pool^1,2^. Under certain stress conditions, such as chemotherapy, HSCs are activated, enter the cell cycle and differentiate to replenish the haematopoietic system^3^. Interferon (IFN), G-CSF, IL-1 signalling and pathogens have key roles in regulating HSC quiescence and activation^4–7^. However, how inflammation is activated in HSCs after a challenge is not fully understood. The innate immune receptor MDA5 belongs to the family of retinoic-acid-inducible gene I (RIG-I)-like receptors and, after its activation, induces inflammation by activating type-I interferons and proinflammatory cytokines^8^. Thus, MDA5 could potentially have a role in HSC activation in response to stress. Nevertheless, the canonical trigger for MDA5 activation is viral RNA that should not exist in HSCs in non-infectious conditions. Thus, an endogenous ligand could activate MDA5 in HSCs. Recently, it has been established that TE transcripts and other endogenous ligands can bind to MDA5 (refs. ^8,9^). For example, *Alu* (non-autonomous retrotransposon TE family) transcripts can bind to and activate MDA5 when the RNA-editing enzyme ADAR is absent^10–12^. Upregulation of TEs by demethylating drugs also activates MDA5 (refs. ^13,14^). Depending on their transposition mechanisms, TEs can be classified into DNA transposons or retrotransposons that can be further separated into long terminal repeat elements (LTRs), such as endogenous retroviruses, or non-LTR elements, such as long interspersed nuclear elements (LINEs) and short interspersed nuclear elements (SINEs). Each of these subclasses contains several TE superfamilies that consist of numerous TE families that harbour tens to thousands of copies^15^. TEs are not only a source of mutation^16^ through transposition, but are also activated by various stress signals^17–19^. In the haematopoietic system, ageing and irradiation lead to the expression of diverse TE families in HSCs^20,21^. TE transcripts present during stress could therefore activate MDA5 to induce inflammatory signalling that is necessary for HSCs to exit quiescence.

In this Article, we show that, during haematopoietic regeneration after chemotherapy, increased expression of TEs induces activation of the innate immune receptor MDA5. Subsequently, MDA5 signalling leads to an inflammatory response that is crucial for HSCs to exit quiescence and proliferate.

## Results

### Inflammatory signalling is activated in HSCs following chemotherapy

**Extended Data Fig. 1:**
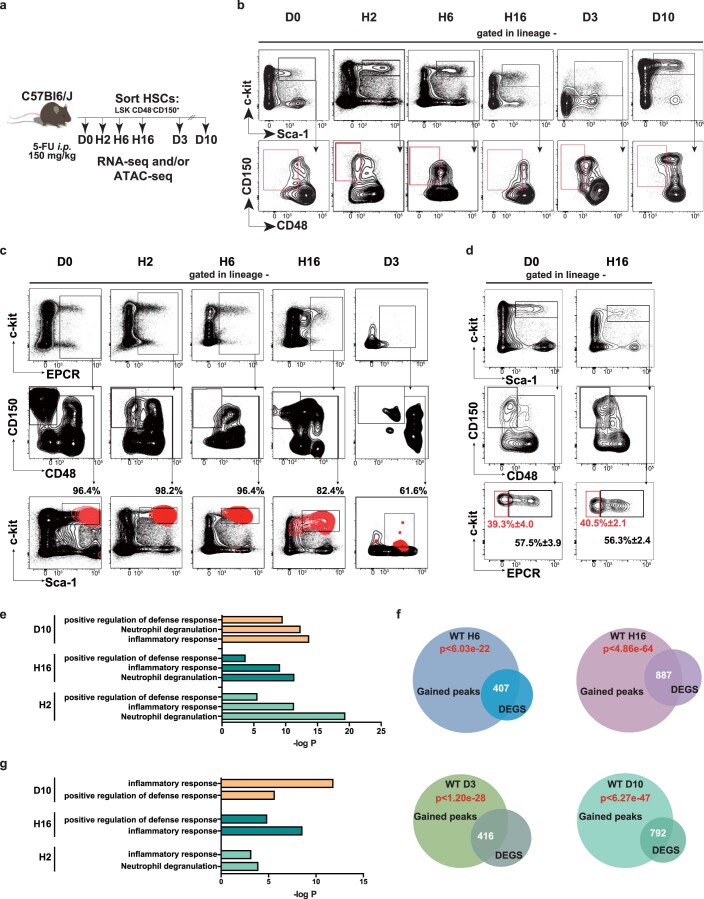
HSC isolation after chemotherapy. **a**, Schematic of the experimental strategy followed for the RNA-seq and ATAC-seq experiments on WT HSCs. **b**, Gating strategies for sorting HSCs from the BM of D0 or 5-FU-injected (H2, H6, H16, D3, D10) mice (15 biologically independent samples- representative plots are shown). **c**, Comparison of our sorting strategy (LSK/SLAM) to the HSCs sorted using EPCR/SLAM (EPCR^+^CD48^−^CD1450^+^) markers. The EPCR/SLAM HSCs are then projected on the LSK/SLAM gating strategy (red color) and the percentage of EPCR/SLAM HSCs that are included in the LSK/SLAM gate is indicated (2 biologically independent samples- representative plots are shown). **d**, Comparison of the number of cells in the LSK/SLAM gate that are not EPCR/SLAM at D0 and H16 (2 biologically independent samples- representative plots are shown). **e**, Gene ontology analysis of the genes upregulated at H2, H16 and D10 after 5-FU injection compared to D0 in WT HSCs. X-axis depicts -logP. **f**, Venn diagrams depicting the overlap of differentially expressed genes (DEGs) in WT HSCs with genes assigned to newly accessible regions-gained ATAC peaks at the indicated time points compared to D0 (-100/+25 kb from TSS, p-values represent hypergeometric test). **g**, Gene ontology analysis of deregulated genes that also exhibit changes in chromatin accessibility at the indicated time points. X-axis depicts -logP.

To understand the molecular mechanisms that govern haematopoietic regeneration, we performed RNA-sequencing (RNA-seq) analysis of HSCs from C57Bl/6J wild-type (WT) mice treated with the myeloablative agent 5-fluoruracil (5-FU). This treatment eliminates all cycling cells and forces HSCs to exit quiescence and proliferate to replenish the bone marrow (BM) cells^22,23^. We sorted HSCs (lineage^−^Sca-1^+^c-Kit^+^CD48^−^CD150^+^, also known as LSK/SLAM, referred to as HSCs in this manuscript) at homeostatic conditions (day 0 (D0)), as well as at 2 h, 6 h and 16 h (H2, H6 and H16, respectively), 3 d (D3, proliferation start^23^) and 10 d (D10) after 5-FU injection (Extended Data Fig. 1a,b). Sorting EPCR/SLAM HSCs^23^ to avoid the change in c-kit expression after chemotherapy showed that more than 90% of EPCR/SLAM cells fall within the LSK/SLAM gate at D0, H2 and H6 and more than 80% at H16 (Extended Data Fig. 1c). The percentage of EPCR^−^ cells in the LSK/SLAM gating was similar from D0 to H16 (Extended Data Fig. 1d).

**Fig. 1:**
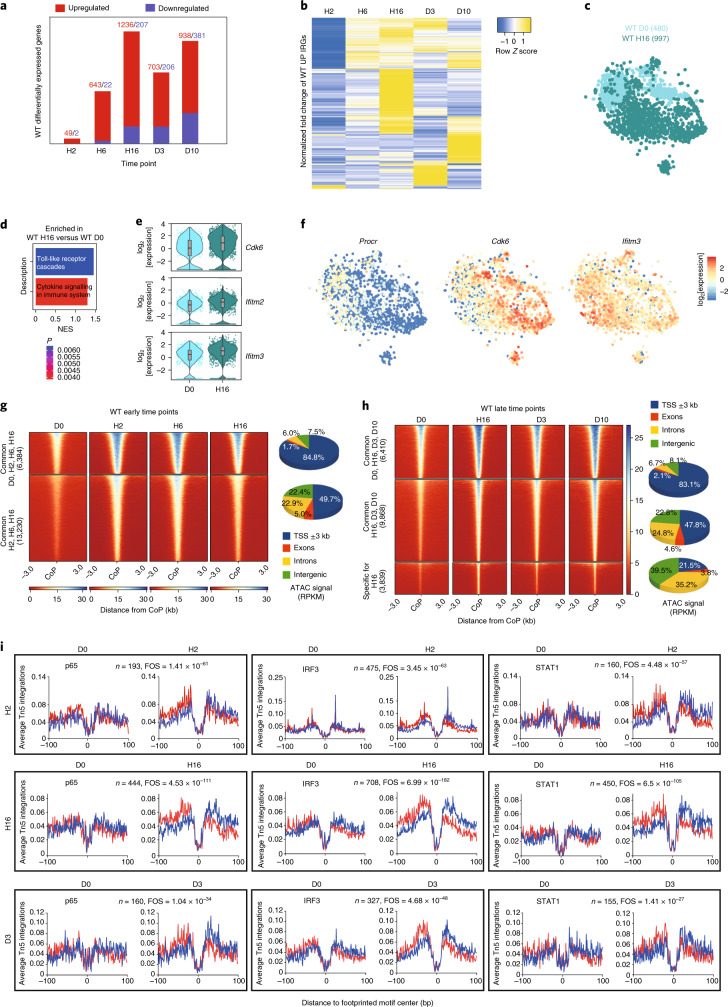
5-FU treatment results in the upregulation of inflammatory signalling in HSCs. **a**, The number of differentially expressed genes at different time points after 5-FU treatment in WT HSCs (LSK/SLAM). *n* = 2 (H2, H6 and D3) and *n* = 3 (D0, H16 and D10) biologically independent samples. Fold change cut-off = 1.5. *P*_adj_ < 0.05. **b**, Heat map of the normalized fold change in the union of IRGs upregulated in WT HSCs at the indicated time points compared to D0. Fold change cut-off = 1.5. *P*_adj_ < 0.05 in at least one time point. **c**, *t*-Distributed stochastic neighbour embedding (*t*-SNE) representation showing sorted WT HSCs at D0 in cyan and at H16 in green (the number of sequenced cells is shown in parentheses). **d**, GSEA of differentially expressed genes among D0 and H16 WT HSCs from **c**. NES, normalized enrichment score. **e**, The log_2_-transformed fold change in expression of the indicated genes at D0 or H16 in WT HSCs from **c**. The boxes show the interquartile range, the whiskers show the minimum and maximum values, and the horizontal line shows the median value. Each dot represents a single cell and the shape of the plot represents probability density. *n* = 480 (WT D0) and *n* = 997 (H16) cells. One independent experiment per time point. *P*_adj_ < 0.05. **f**, *t*-SNE representation showing the expression of differentially expressed genes among H16 and D0 in WT HSCs. The colour scale represents the log_2_-transformed normalized transcript counts. **g**,**h**, Heat maps (left) of the differentially accessible regions in WT HSCs at the indicated early (**g**) and late (**h**) time points ±3 kb from the centre of the peak (CoP). Right, the genomic location distribution of the accessible regions. **i**, Average normalized Tn5 insertion profiles around footprinted motifs (p65, IRF3, STAT1) in merged ATAC peaks at the indicated time points after 5-FU treatment in WT HSCs. Footprint numbers (*n*) are indicated at the top. Footprint occupancy scores (FOS) indicate the significance versus D0. Insertions on the sense and antisense DNA strands are indicated in red and blue, respectively.

We analysed the transcriptional response during the 5-FU challenge by comparing the RNA-seq data between D0 and all of the consecutive time points. Few deregulated genes were observed at H2, but they increase throughout the time course and many genes remain deregulated even at D10 after treatment (51 (H2), 1,443 (H16), 1,319 (D10) deregulated genes; fold change cut-off = 1.5; *P*_adj_ < 0.05; Fig. 1a and Supplementary Tables 1–5). Gene ontology (GO) analysis showed that ‘inflammatory response’ was enriched in upregulated genes from H2 to D10 (Extended Data Fig. 1e). Given the important role of inflammatory signalling in HSCs^4,5,24^, we identified the interferon-regulated genes (IRGs) from the Interferome database (http://interferome.org)^25^ and found upregulation of IRGs, especially at H16 and a second wave at D10 (Fig. 1b). We also sequenced 480 WT single HSCs from D0 and 997 from H16 (Fig. 1c). Gene set enrichment analysis (GSEA) showed enrichment for Toll-like receptor and cytokine signalling at H16 (Fig. 1d). The HSC marker *Procr* (also known as *Epcr)* was highly expressed in D0 HSCs, while the activation marker *Cdk6* and IRGs such as *Ifitm2* and *Ifitm3* were highly expressed in HSCs at H16 (Fig. 1e,f and Supplementary Table 6). Collectively, our results at the bulk and single-cell level show that inflammation-related genes are activated after chemotherapy.

### Chemotherapy leads to chromatin reorganization in HSCs

To examine changes in chromatin accessibility during chemotherapy, we performed an assay for transposase-accessible chromatin followed by sequencing (ATAC-seq) at the same time points after chemotherapy. By comparing the early timepoints H2, H6 and H16 with D0, we found ~6,000 common accessible regions, mostly spanning transcriptional start sites (TSS), and ~13,000 regions gaining accessibility and spanning TSS, introns and intergenic regions (Fig. 1g and Supplementary Tables 7–11). Some regions also lost accessibility. By comparing H16, D3 and D10 with D0, we found ~4,000 regions that are uniquely accessible at H16 and lose accessibility by D3 (Fig. 1h). We assigned the differential ATAC-seq peaks to genes (−100 kb/+25 kb from the TSS) and identified a significant overlap, with the differentially expressed genes at all of the time points except for H2 showing enrichment for inflammatory response genes (Extended Data Fig. 1f,g). By performing digital footprinting analysis to identify motif occupancy for transcription factors, we observed increased occupancy for IRF3, NF-κB (p65) and STAT1 at H2, which peaked at H16 and was less prominent at D3 after chemotherapy (Fig. 1i). Our genome-wide analysis shows that, after myeloablative stress, changes in chromatin accessibility are observed in HSCs.

### TE family expression is increased during haematopoietic regeneration

**Fig. 2:**
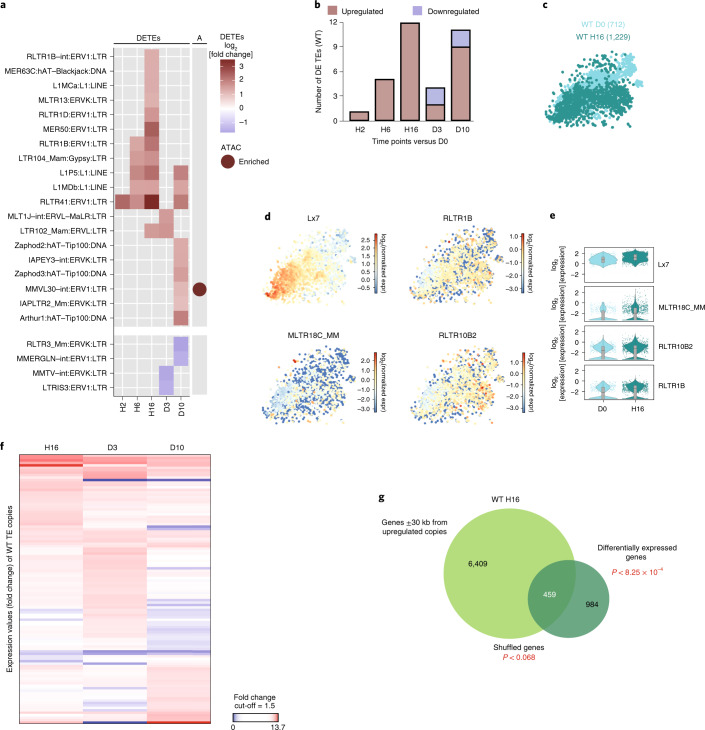
Rapid TE upregulation in HSCs after 5-FU treatment. **a**, Heat map of the log_2_-transformed fold change of differentially expressed TE families (DETEs) detected in WT HSCs at the indicated time points after 5-FU treatment. TE families that have a significantly enriched ATAC-seq peak nearby (±1 kb) are highlighted in the right column (A). **b**, The number of upregulated or downregulated TE families at the indicated time points after 5-FU treatment. **c**, *t*-SNE representation of sorted WT HSCs (LSK/SLAM) at D0 (green) and H16 (dark green) (the number of sequenced cells is shown in parentheses). **d**, *t*-SNE representation showing the expression of differentially expressed TE families between H16 and D0 in WT HSCs. The colour scale represents the log_2_-transformed normalized transcript counts. **e**, The log_2_-transformed fold change in expression of the TE families in **d** at D0 or H16 in WT HSCs from **c**. The box shows the interquartile range, the whiskers show the minimum and maximum values, and the horizontal line shows the median value. Each dot represents a single cell and the shape of the plot represents probability density. *n* = 712 (WT D0) and *n* = 1,229 (H16) cells. One independent experiment per time point. *P*_adj_ < 0.05. **f**, Heat map of the expression values (fold change) of TE copies in WT HSCs at the indicated time points compared to D0. Fold change cut-off = 1.5. *P*_adj_ < 0.05. **g**, The overlap between genes in proximity (±30 kb from TSS of the genes) to upregulated TE copies in WT HSCs and deregulated genes at H16 (*P* < 8.25 × 10^−4^). The *P* value of the control overlap after gene shuffling is also shown (*P* < 0.068).

We next wondered whether chromatin reorganization affects the expression of the repetitive part of the genome and investigated the expression of TE families by mapping the RNA-seq reads using STAR^26^, and quantifying TE family expression using TEtranscripts^27^ (multi-mapped reads). Upregulation of TE families was observed. Indeed, the RLTR41:ERV1:LTR family is already upregulated at H2, whereas 5 and 12 families are upregulated at H6 and H16, respectively (fold change cut-off = 1.5; *P*_adj_ < 0.05). This increase in expression is progressive as the families upregulated at H2 and H6 remain upregulated at H16 (Fig. 2a,b and Supplementary Table 12). At D10, nine families are upregulated in total and six of them are new, concomitant to a second wave of IRGs. Downregulation of two families occurs at D3 and D10 (Fig. 2a,b). The majority of deregulated TEs belong to the LINE1, ERV1 and ERVK families (Fig. 2a). We next intersected the uniquely mapped ATAC-seq peaks (Supplementary Table 13) with TE copies (±1 kb to increase mapping). We searched for TE families that were enriched in ATAC-seq, but also deregulated after 5-FU treatment to pinpoint specific families suffering chromatin changes and consequent transcriptional activation. Only one upregulated TE family, MMVL30-int:ERV1:LTR, was enriched in newly accessible chromatin regions (Fig. 2a). Upregulated TE families were also identified at the single-cell level, albeit differences were observed between bulk and single-cell RNA-seq (Fig. 2c–e and Supplementary Table 14). These results confirm that TE families are upregulated after chemotherapy.

We next examined whether deregulation of TE family expression is due to deregulation of several or specific copies within a family. We filtered for uniquely mapping RNA-seq reads and unravelled specific TE copies with expression changes after 5-FU treatment at H16, D3 and D10. This analysis should be taken with caution, as many recent and potentially active TE copies will not be included due to mapping issues. We found that 37 TE copies were upregulated at H16, 42 at D3 and 37 at D10, which also showed significant downregulation of many TE copies (fold change cut-off 1.5; *P*_adj_ < 0.05; Fig. 2f and Supplementary Table 15). We then examined whether the deregulated TE copies were proximal to deregulated genes^17^. We identified the genes proximal to upregulated TE copies (±30 kb from the gene TSS). As a control, we searched TE–gene pairs using a random list of genes. The percentages of deregulated TE–gene pairs between observed and expected were then compared and the result was significant for H16 (Fig. 2g). Thus, few copies were confidently detected as upregulated after chemotherapy and some of these copies are proximal to deregulated genes.

### TE transcripts could act as MDA5 ligands after stress

**Extended Data Fig. 2:**
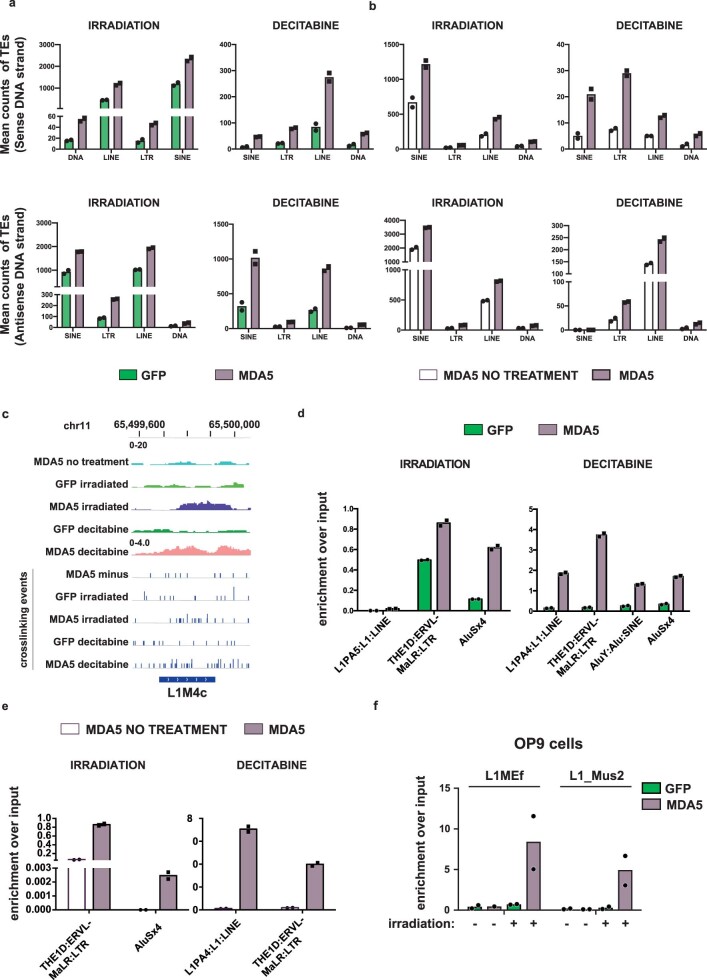
TEs bind to MDA5 upon stress in human and mouse cells. **a**, Bar graphs depicting the mean counts of LINE, SINE, LTR and DNA transposon (DNA) RNA (fold change >1.5 and p-value < 0.05) bound to MDA5 or GFP after irradiation or decitabine treatment (sense DNA strand-upper panel, antisense DNA strand-lower panel) (n = 2 biologically independent population samples, 2 independent experiments). **b**, Bar graphs depicting the mean counts of LINE, SINE, LTR and DNA transposon (DNA) RNA (fold change >1.5 and p-value < 0.05) bound to MDA5 after irradiation or decitabine treatment or to MDA5 without treatment (sense DNA strand-upper panel, antisense DNA strand-lower panel) (n = 2 biologically independent population samples, 2 independent experiments). **c**, Representative track that shows binding of L1M4c to GFP after irradiation or decitabine treatment and to MDA5 without treatment or after irradiation or decitabine treatment. Y-axis represents RPKM. Crosslinking events are also shown. **d-e** qPCR experiments after FLASH depicting binding of TEs to GFP or MDA5 after irradiation or decitabine treatment **(d)** or to MDA5 without treatment or after irradiation or decitabine treatment **(e)** (n = 2 biologically independent samples and experiments). **f**, qPCR experiment after FLASH from mouse OP9 cells depicting binding of LINE1 elements to GFP or MDA5 without treatment or after irradiation (n = 2 biologically independent samples and experiments). Source data

We next hypothesized that upregulated TE transcripts could activate the innate immune receptor MDA5 leading to the inflammatory signalling observed during regeneration. To determine whether TE transcripts bind to MDA5 in response to stress, we performed fast ligation of RNA after some sort of affinity purification for high-throughput sequencing (FLASH)^28^ analysis in HEK293 human embryonic kidney cells overexpressing green fluorescent protein (GFP) or MDA5. Irradiation was used as a stress signal and the methyltransferase inhibitor decitabine was used as a positive control^29^. We observed binding of MDA5 to RNA of SINEs, LINEs, LTRs, some DNA transposons and mitochondrial RNAs, consistent with previous reports^10,13,14,30^ (Supplementary Tables 16 and 17). Overall, after irradiation or decitabine treatment, binding of TE transcripts to MDA5 was higher than binding to control GFP protein (Extended Data Fig. 2a,c), or to MDA5 in the absence of any treatment (Extended Data Fig. 2b,c). Notably, some coding genes and other RNAs can bind to MDA5. These results were confirmed by qPCR after ultraviolet cross-linking for a panel of TEs in HEK293 cells, but also in mouse OP9 stromal cells (Extended Data Fig. 2d–f). These results show that TE transcripts could bind to MDA5 after stress.

### *Mda5*^−/−^ HSCs are resistant to activation and have a better repopulation capacity

**Fig. 3:**
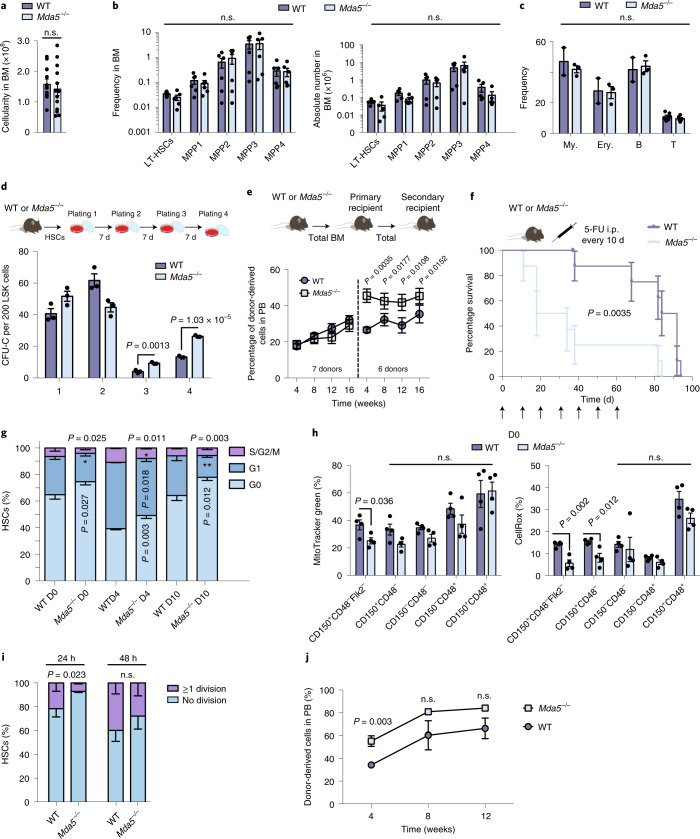
MDA5 is required for HSC activation. **a**, The BM cellularity of WT or *Mda5*^−/−^ mice. *n* = 13 biologically independent samples. Data are mean + s.d. Statistical analysis was performed using two-tailed *t*-tests. **b**, The frequency (left) and the absolute numbers (right) of LT-HSCs, and MPPs from BM of WT or *Mda5*^−/−^ mice. *n* = 6 (BM frequency) and *n* = 5 (absolute numbers) biologically independent samples. Data are mean + s.d. Statistical analysis was performed using two-tailed *t*-tests. **c**, The frequency of myeloid (My; CD11^+^Gr1^+^), erythroid (Ery; Ter119^+^), B cells (B220^+^) in the BM and T cells (CD3^+^) in the thymus. For myeloid, erythroid and B cells, *n* = 2 (WT) and *n* = 3 (*Mda5*^−/−^); and, for T cells, *n* = 6 biologically independent samples. Data are mean + s.d. Statistical analysis was performed using two-tailed *t*-tests. **d**, Serial CFU-C assay of BM HSCs from WT or *Mda5*^−/−^ mice scored every 7 d. *n* = 3 biologically independent samples. Data are mean ± s.d. Statistical analysis was performed using two-tailed *t*-tests. **e**, The percentage of donor-derived cells in peripheral blood (PB) of primary and secondary recipients in weeks after injection. The dotted line separates the primary from secondary transplantation. *n* = 30 (primary) and *n* = 15 (secondary) biologically independent samples, with *n* = 4 and *n* = 3 independent experiments, respectively. Data are mean ± s.e.m. Statistical analysis was performed using two-tailed *t*-tests. **f**, Kaplan–Meier survival curve of WT or *Mda5*^−/−^ mice after 5-FU injections every 10 d. *n* = 8 mice. Statistical analysis was performed using the log-rank (Mantel–Cox) test. **g**, Cell cycle status of WT or *Mda5*^−/−^ HSCs after 5-FU treatment. For WT, *n* = 8 (D0), *n* = 5 (D4) and *n* = 4 (D10); and, for *Mda5*^−/−^, *n* = 9 (D0), *n* = 5 (D4) *n* = 6 (D10) biologically independent samples. Data are mean ± s.d. Statistical analysis was performed using two-tailed *t*-tests. **h**, The frequency of cells with detectable mitochondrial mass (left) and ROS (right) at D0. *n* = 4 biologically independent samples. Data are mean + s.d. Statistical analysis was performed using two-tailed *t*-tests. **i**, The percentage of HSCs (LSK/SLAM, Flk2^+^) that had undergone at least one division or no division after 24 h or 48 h. *n* = 3 biologically independent samples. Data are mean ± s.d. Statistical analysis was performed using two-tailed *t*-tests. **j**, The percentage of donor-derived cells in the peripheral blood of primary recipients transplanted with either WT or *Mda5*^−/−^ HSCs cultured for 48 h. *n* = 5 biologically independent samples. Data are mean ± s.e.m. Statistical analysis was performed using two-tailed *t*-tests. n.s., not significant. Source data

**Extended Data Fig. 3:**
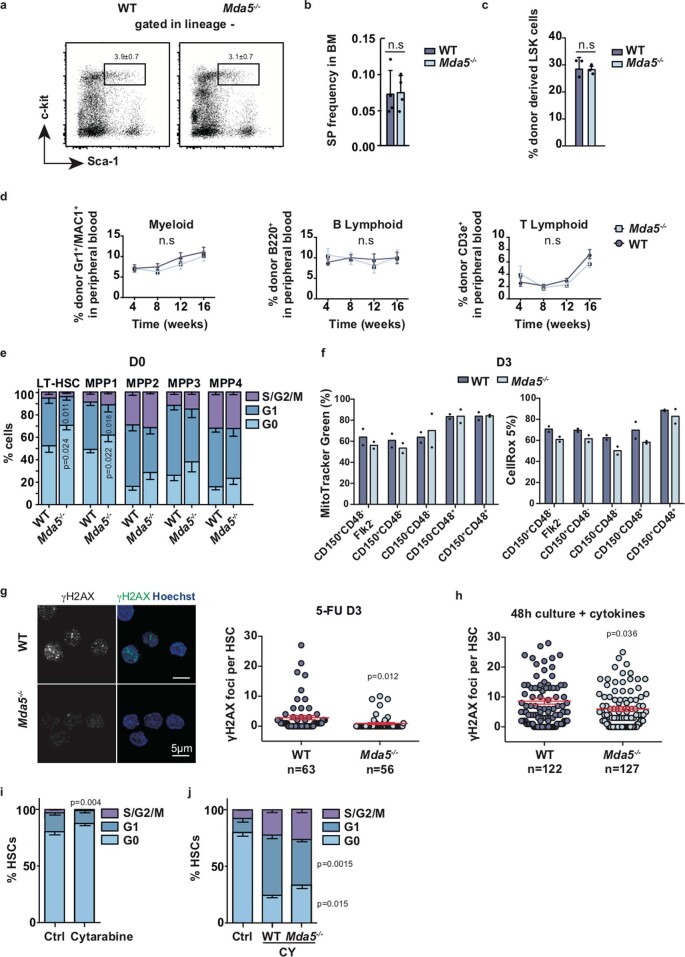
MDA5 is required for HSC activation. **a**, Representative profile comparing Sca-1 expression on the lineage negative fraction of the BM of WT or *Mda5*^-/-^ mice (3 biologically independent samples, one representative plot is shown). **b**, Side population (SP) frequency in the BM of WT or *Mda5*^-/-^ mice (n = 4 biologically independent samples, mean+s.d, *two-tailed t-test*, n.s. non-significant). **c**, Homing assay: percentage of donor derived LSK cells in the BM of WT recipients (n = 3 biologically independent samples) 16hrs after injection of BM cells from WT or *Mda5*^-/-^ mice (mean + s.d., *two-tailed t-test*, n.s. non-significant). **d**, Percentage of donor derived myeloid, B or T lymphoid cells in the peripheral blood of recipients injected with BM cells isolated from WT (n = 30 biologically independent samples) or *Mda5*^-/-^ mice (n = 27 biologically independent samples). Time (weeks) denotes the time after intravenous injection (mean ± s.d, *two-tailed t-test*, n.s. non-significant). **e**, Cell cycle analysis of HSCs and MPPs as indicated (n = 7 biologically independent samples, mean-s.d., *two-tailed t-test*). **f**, Bar graphs depicting the frequency of cells with detectable mitochondrial mass measured by MitoTracker Green (left panel) and reactive oxygen species (ROS) production (right panel) at D3 after 5-FU injection (n = 2 biologically independent samples and experiments). **g**, Images of γH2AX foci positive HSCs from WT or *Mda5*^-/-^ mice (left) and quantification of γH2AX foci per nuclei at D3 after 5-FU injection (mean ± s.e.m., *two-tailed t-test*, n = 64-WT and 56-*Mda5*^-/-^ cells examined in 2 independent experiments). **h**, Dot plot representing quantification of γH2AX foci per WT or *Mda5*^-/-^ HSCs nuclei quantified with Imaris software 9.2 after culturing cells for 48 h (n = 121-WT and n = 127-*Mda5*^-/-^ cells examined in 2 independent experiments, mean±s.e.m., *two-tailed t-test*, *P = 0.036). **i**, Cell cycle analysis of WT HSCs after cytarabine treatment (n = 4 biologically independent samples and experiments, mean-s.d., *two-tailed t-test*). **j**, Cell cycle analysis of WT and *Mda5*^-/-^ HSCs after cyclophosphamide (n = 7 biologically independent samples and experiments, mean-s.d., *two-tailed t-test*). Source data

We reasoned that, if MDA5 has a functional role in HSC activation, ablation of MDA5 should have consequences in HSC biology. We examined the function of MDA5 in HSCs using *Ifih1*-knockout (also known as *Mda5*; hereafter *Mda5*^−/−^; B6.Cg-*Ifih1*tm1.1Cln/J)^31^. We analysed BM cellularity, the frequency and absolute numbers of HSCs, multipotent progenitors (MPP1–4) and differentiated haematopoietic populations, and found no significant differences between the knockout and WT mice under homeostatic conditions (Fig. 3a–c). As *Mda5* is an IRG, we confirmed that SCA-1 expression was not affected in *Mda5*^−/−^ HSCs and that the frequency of HSCs in the BM using the side population^32^ remained unchanged (Extended Data Fig. 3a,b). Thus, MDA5 does not interfere with the haematopoietic composition of the BM under steady-state conditions.

To determine HSC clonogenic activity, we sorted HSCs from WT and *Mda5*^−/−^ mice and performed colony-forming-unit-cell (CFU-C) replating assays. *Mda5*^−/−^ HSCs produced more CFU-C colonies after the third and fourth plating compared with WT HSCs (Fig. 3d). Competitive in vivo transplantation assays showed no significant differences in primary transplantations (Fig. 3e). Homing and contribution to myeloid and lymphoid lineages was similar between WT and *Mda5*^−/−^ HSCs (Extended Data Fig. 3c,d). The levels of chimerism were higher in secondary recipients that were transplanted with *Mda5*^−/−^ HSCs compared with in those that were injected with WT HSCs (Fig. 3e). However, after serial 5-FU injections every 10 d, the *Mda5*^−/−^ mice died significantly earlier than WT mice (Fig. 3f). These results imply that *Mda5*^−/−^ HSCs may be more quiescent compared with WT HSCs, therefore performing better in the long-term; however, during rapid acute stress such as serial 5-FU injections, *Mda5*^−/−^ mice are not able to reconstitute their blood system fast enough.

Next, we examined the cell cycle status of *Mda5*^−/−^ HSCs. When compared to the WT, the BM of *Mda5*^−/−^ mice had significantly more quiescent (cells in G0) HSCs after treatment with 5-FU, but also at steady state (Fig. 3g and Extended Data Fig. 3e). This steady-state phenotype was also significant for MPP1 cells, but not for other progenitors (Extended Data Fig. 3e). *Mda5*^−/−^ HSCs also had lower mitochondrial mass and reactive oxygen species (ROS) levels compared with their WT counterpart at D0, but not at D3 (Fig. 3h and Extended Data Fig. 3f). By examining γH2AX foci as a biomarker for HSC activation, we detected fewer γH2AX foci in *Mda5*^−/−^ HSCs compared with in WT HSCs at D3 after 5-FU injection or after culture, consistent with their ability to remain in G0 state (Extended Data Fig. 3g,h). Furthermore, the percentage of HSCs undergoing at least one division was decreased after 24 h and 48 h (although not significant in the latter) when cultured ex vivo (Fig. 3i). We also transplanted WT or *Mda5*^−/−^ HSCs that remained in culture for 48 h and observed that animals receiving *Mda5*^−/−^ HSCs exhibited higher chimerism levels (Fig. 3j). Finally, we checked whether the lack of MDA5 impairs HSC activation after the use of other chemotherapeutics, namely cytarabine and cyclophosphamide. Cytarabine treatment, at least at the dose that we used, could not drive HSC cycling, but cyclophosphamide treatment led to HSC cycling and this function was impaired in *Mda5*^−/−^ HSCs (Extended Data Fig. 3i,j). Together these results suggest that HSCs lacking MDA5 exhibit impaired exit from quiescence during regenerative stress.

### Chemotherapy induces TE transcription in *Mda5*^−/−^ HSCs

**Fig. 4:**
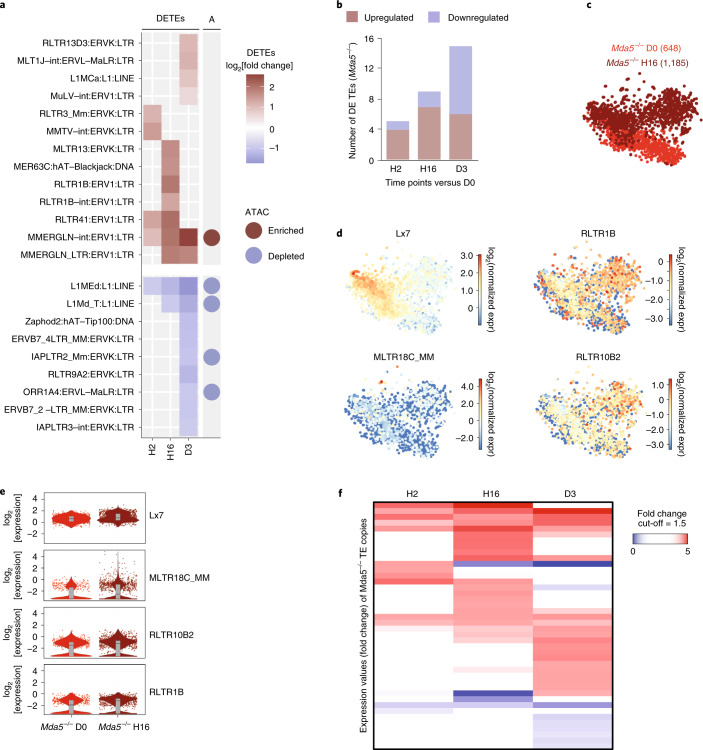
TE upregulation in *Mda5*^−/−^ HSCs after chemotherapy. **a**, Heat map of the log_2_-transformed fold change of all differentially expressed TE families detected in *Mda5*^−/−^ HSCs at the indicated time points after 5-FU treatment. TE families that had a significantly enriched or depleted ATAC-seq peak nearby (±1 kb) are highlighted in the right column (A). **b**, The number of upregulated or downregulated TE families in *Mda5*^−/−^ HSCs at the indicated time points after 5-FU treatment. **c**, *t*-SNE representation of sorted *Mda5*^−/−^ HSCs (LSK/SLAM) at D0 (red) and H16 (dark red) (the number of sequenced cells is indicated in parentheses). **d**, *t*-SNE representation showing the expression of differentially expressed TE families between H16 and D0 in *Mda5*^−/−^ HSCs. The colour scale represents the log_2_-transformed normalized transcript counts. **e**, The log_2_-transformed fold change in expression of the TE families shown in **d** at D0 or H16 in *Mda5*^−/−^ HSCs from **c**. The box shows the interquartile range, the whiskers show the minimum and maximum values, and the horizontal line shows the median value. Each dot represents a single cell and the shape of the plot represents probability density. *n* = 648 (D0) and *n* = 1,185 (H16) *Mda5*^−/−^ cells. One independent experiment per time point. *P*_adj_ < 0.05. **f**, Heat map of the expression values (fold change) of TE copies in *Mda5*^−/−^ HSCs at the indicated time points compared to D0. Fold change cut-off = 1.5. *P*_adj_ < 0.05.

We next reasoned that *Mda5*^−/−^ HSCs retain their quiescence either because TEs are not upregulated or because the activation of inflammatory signalling is impaired in *Mda5*^−/−^ mice. TE families were indeed deregulated in *Mda5*^−/−^ HSCs; 4 families were upregulated at H2, 7 at H16 and 6 at D3, belonging mostly to LINE1, ERVK and ERV1 families (Fig. 4a and Supplementary Table 18). Five out of the seven upregulated families at H16 were also upregulated for the WT. However, downregulation of TE families was also observed in *Mda5*^−/−^ HSCs, especially at D3 (Fig. 4a). One family, MMERGLN-int:ERV1:LTR, was enriched in newly accessible chromatin regions, but four of the downregulated families were enriched in regions that lost accessibility (Fig. 4a). At the single-cell level, TE families that were upregulated in the single-cell RNA-seq analysis of WT HSCs were also upregulated in *Mda5*^−/−^ HSCs (Fig. 4b–e and Supplementary Table 19). Furthermore, 9 TE copies were upregulated at H2, 16 at H16 and 17 at D3, indicating that less TE copies were upregulated in comparison to WT HSCs (Fig. 4f and Supplementary Table 20). The proximity of TE copies to genes was not significant at any time point. Thus, it is possible that MDA5 has a role in TE upregulation. Collectively, TEs are upregulated in *Mda5*^−/−^ HSCs following similar patterns to WT HSCs, albeit with some differences.

### Inflammatory signalling is impaired in *Mda5*^−/−^ HSCs after chemotherapy

**Fig. 5:**
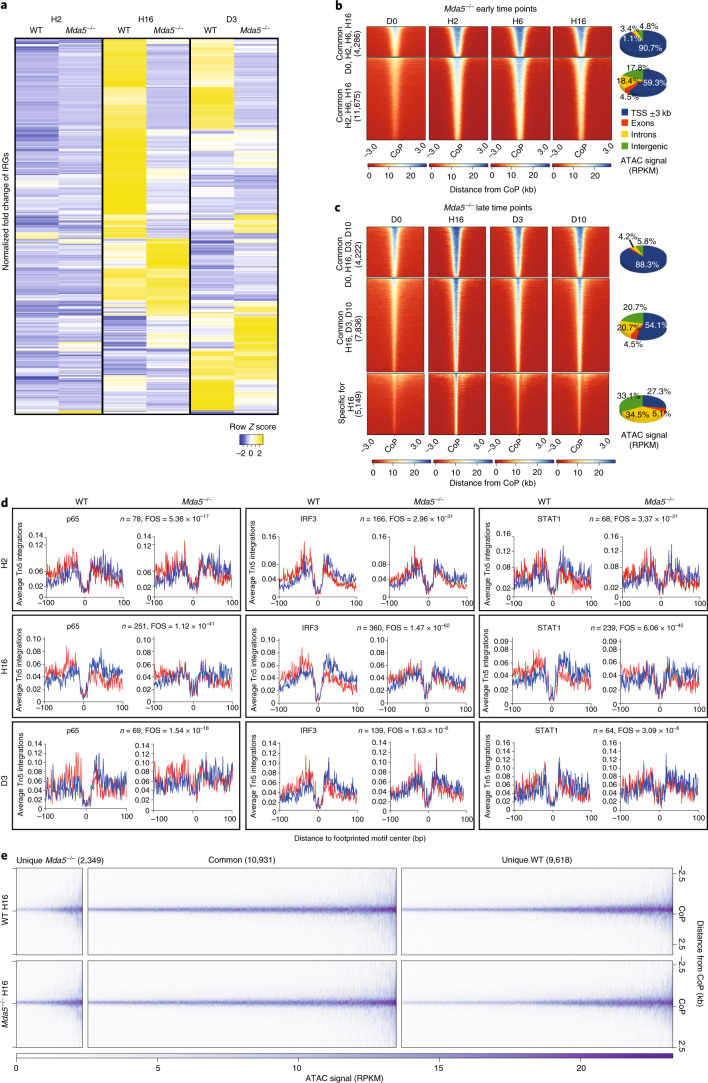
Impaired inflammatory signalling in *Mda5*^−/−^ HSCs. **a**, Heat map of the normalized fold change in the union of IRGs upregulated in control WT or *Mda5*^−/−^ HSCs at the H2, H16 or D3 time points compared to D0. Fold change cut-off = 1.5. *P*_adj_ < 0.05, at least at one time point. **b**,**c**, Heat map (left) of the common and differentially accessible regions in *Mda5*^−/−^ HSCs at D0, H2, H6 and H16 (**b**) or at D0, H16, D3 and D10 (**c**) ±3 kb from the centre of the peak. Right, the genomic location distribution of the accessible regions in each cluster of the heat map. **d**, Average normalized Tn5 insertion profiles around footprinted motifs (p65, IRF3, STAT1) in merged ATAC peaks at the indicated time points after 5-FU treatment in WT or *Mda5*^−/−^ HSCs. Footprint numbers (*n*) are indicated at the top. Footprint occupancy scores indicate significance versus D0. Insertions on the forward and reverse DNA strands are indicated in red and blue, respectively. **e**, Heat map of common and differentially accessible regions in WT and *Mda5*^−/−^ HSCs at H16.

**Extended Data Fig. 4:**
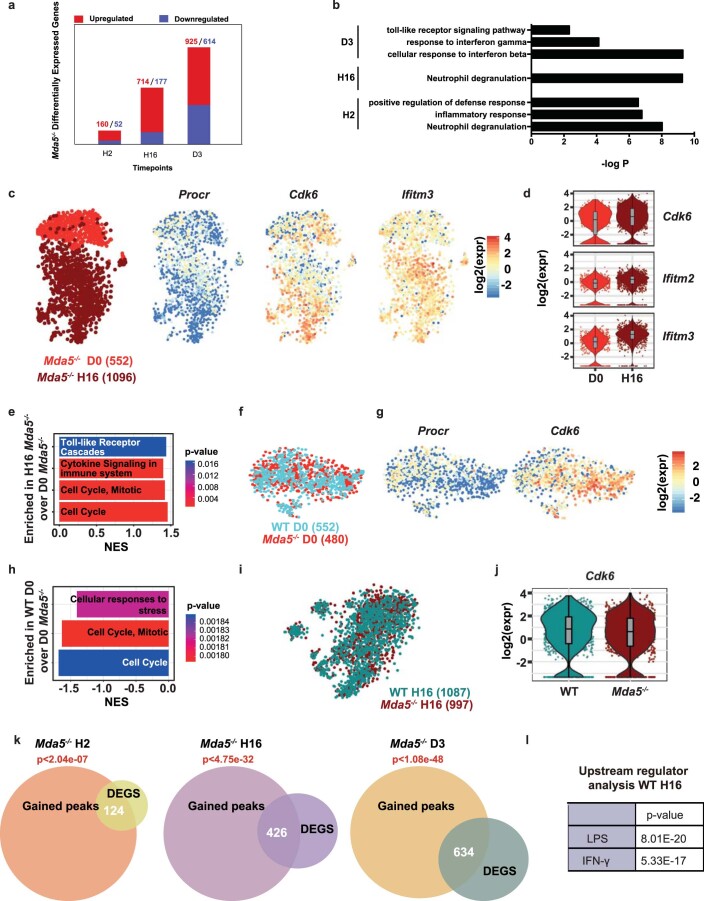
5-FU treatment in *Mda5*^*-/-*^ HSCs. **a**, Bar graphs depicting the number of differentially expressed genes at different time points after 5-FU treatment in *Mda5*^-/-^HSCs (H2, H16: n = 2 and D0, D3: n = 3 biologically independent samples, fold change cut-off 1.5, *Padj* < 0.05). **b**, Gene ontology of upregulated genes at indicated time points versus D0. **c**, t-SNE representation of *Mda5*^-/-^ HSCs at D0 (red) and H16 (dark red) (left) (number of sequenced cells in parentheses). t-SNE representation of DEGs between H16 and D0 in *Mda5*^-/-^ HSCs. Color scale: log2 of normalized transcript counts (right) **d**, Violin plots depicting log2 fold change expression at D0 or H16 in *Mda5*^-/-^ HSCs. Box: interquartile range, whiskers: minimum and maximum values, horizontal line: median. Each dot represents a single cell; the plot shape declares probability density (n = 552 *Mda5*^-/-^ D0 and n = 1096 H16 cells, one independent experiment per time point, *Padj* < 0.05). **e**, Gene set enrichment analysis in *Mda5*^-/-^ HSCs from **c** between D0 and H16 **f**, t-SNE representation of WT (blue) and *Mda5*^-/-^ HSCs (red) at D0 (number of sequenced cells in parentheses). **g**, t-SNE representation of DEGs between H16 and D0 in WT and *Mda5*^-/-^ HSCs. Color scale: log2 of normalized transcript counts. **h**, Gene set enrichment analysis between WT and *Mda5*^-/-^ HSCs at D0. **i**, t-SNE representation of WT (green) and *Mda5*^-/-^ HSCs (red) at H16 (number of sequenced cells in parentheses). **j**, Violin plots depicting log2 expression of *Cdk6* at H16 in WT and *Mda5*^-/-^ HSCs. Box: interquartile range, whiskers: minimum and maximum values, horizontal line: median. Each dot represents a single cell; the plot shape declares probability density (n = 1087 WT H16 and n = 997 *Mda5*^-/-^ H16 cells, one independent experiment per time point, *Padj* < 0.05). **k**, Venn diagrams depicting the overlap of differentially expressed genes (DEGs) with genes gaining accessibility (-100/+25 kb from TSS) (p-values: hypergeometric test). **l**, Table of upstream regulators for WT unique accessible regions assigned to proximal genes (+/-25kB) at H16.

Next, we reasoned that an impaired inflammatory response in *Mda5*^−/−^ HSCs would explain their enhanced quiescence. Similar to the WT, gene upregulation was observed while some inflammatory signalling pathways were enriched (fold change cut-off = 1.5; *P*_adj_ < 0.05; Extended Data Fig. 4a,b and Supplementary Tables 21–23). By comparing the expression of *Mda5*^−/−^ HSCs at H16 versus D0 at the single-cell level, we found that inflammatory genes are expressed at higher levels at H16 together with activation markers, such as *Cdk6*, and enrichment for inflammatory signalling pathways was observed (Extended Data Fig. 4c–e). We next compared the WT and *Mda5*^−/−^ HSCs at the single-cell level (Extended Data Fig. 4f,g). GSEA analysis of the whole dataset revealed that cell-cycle-associated genes are enriched in the WT HSCs at D0, while *Cdk6* expression was significantly less in *Mda5*^−/−^ HSCs at H16 (Extended Data Fig. 4h–j and Supplementary Tables 24–26). IRG upregulation in the bulk RNA-seq data was blunted in *Mda5*^−/−^ HSCs in comparison to WT HSCs (Fig. 5a). Thus, transcriptional changes in *Mda5*^−/−^ HSCs compared with WT HSCs suggest that the IFN response is impaired in *Mda5*^−/−^ HSCs.

ATAC-seq assays revealed that, in *Mda5*^−/−^ HSCs, as in WT cells, numerous genomic regions gain accessibility from H2 onwards while some of these regions begin to compact from D3 onwards (Fig. 5b,c and Supplementary Tables 27–31). A significant number of deregulated genes also showed changes in chromatin accessibility at all time points (Extended Data Fig. 4k). Motif occupancy of inflammatory transcription factors in *Mda5*^−/−^ HSCs at H2, H16 and D3 was significantly reduced compared with that of WT HSCs (Fig. 5d). Through comparison of the accessible regions between WT and *Mda5*^−/−^ HSCs at H16, we observed many regions that are unique to either WT or *Mda5*^−/−^ HSCs (Fig. 5e). By assigning these regions to adjacent genes (±25 kb) and performing upstream regulator analysis at the genes adjacent to uniquely accessible regions at WT HSCs, we found regulators such as LPS and IFNγ that were absent when the same analysis was performed for the uniquely accessible regions of *Mda5*^−/−^ HSCs (Extended Data Fig. 4l).

**Fig. 6:**
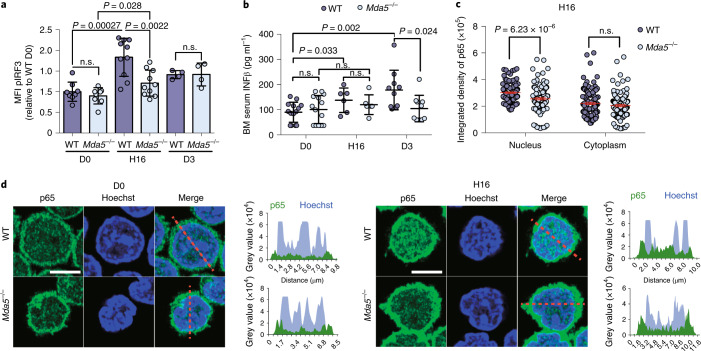
5-FU-induced inflammation is MDA5-dependent. **a**, Relative changes in median fluorescence intensity (MFI) of phosphorylated IRF3 (pIRF3) in WT or *Mda5*^−/−^ HSCs at D0, H16 or D3 after 5-FU treatment, normalized to the WT D0. *n* = 8 biologically independent samples in *n* = 3 independent experiments (D0), *n* = 10 biologically independent samples in *n* = 3 independent experiments (H16) and *n* = 4 biologically independent samples in one experiment (D3). Each dot represents one mouse. Data are mean ± s.d. Statistical analysis was performed using two-tailed *t*-tests; n.s. not significant. **b**, The amount of IFNβ (pg ml^−1^) measured in the BM serum of WT or *Mda5*^−/−^ mice at D0, H16 or D3 after 5-FU treatment. Each dot represents one mouse. *n* = 14 (D0), *n* = 6 (H16), *n* = 10 (D3) biologically independent samples in *n* = 2 (D0 and D3) and *n* = 1 (H16) independent experiments. Data are mean ± s.d. Statistical analysis was performed using two-tailed *t*-tests; n.s., not significant. **c**, The integrated density of the NF-κB subunit p65 in the cytoplasm and the nucleus of WT or *Mda5*^−/−^ HSCs at H16 after 5-FU treatment. *n* = 129 (WT) and *n* = 132 (*Mda5*^−/−^) HSCs examined in *n* = 2 independent experiments. Statistical analysis was performed using two-tailed *t*-tests; n.s., not significant. **d**, Immunostaining for NF-κB subunit p65 in WT or *Mda5*^−/−^ HSCs at D0 and H16 after 5-FU treatment. *n* = 2 independent experiments. Scale bar, 5 μm. The histograms on the right represent the grey value intensity of both p65 (green) and Hoechst (blue) as indicated in the figure by the red dashed line. Source data

**Extended Data Fig. 5:**
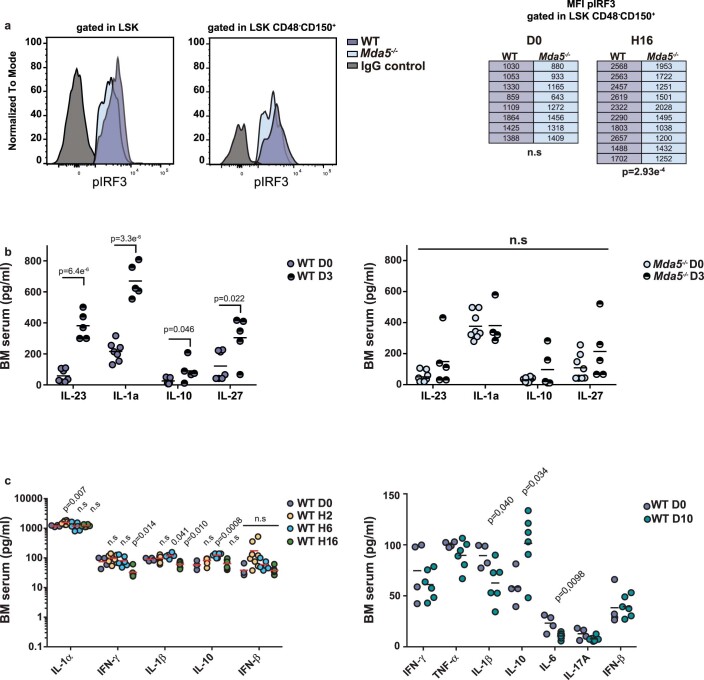
5-FU treatment induces inflammation. **a**, Representative plot depicting phospho-IRF3 staining in WT and *Mda5*^-/-^ HSCs gated in LSK cells or HSCs (left panels). Tables depicting the mean fluorescence intensity (MFI) of pIRF3 in WT and *Mda5*^-/-^ HSCs at the indicated time points (right panels) (*two-tailed t-test*, P values are indicated below). **b**, Measurement of secreted cytokines in WT (left) or *Mda5*^-/-^ (right) BM at D0 and D3 (n = 7 D0 and n = 5 D3 for WT and n = 8 D0 and n = 5 D3 for *Mda5*^-/-^ biologically independent samples, 2 independent experiments, *two-tailed t-test*, n.s: not significant, horizontal line: mean). **c**, Measurement of secreted cytokines in WT bone marrow at D0 (n = 4), H2 (n = 8), H6 (n = 6) and H16 (n = 6) (left) or D0 (n = 4) and D10 (n = 6) (right) (all biologically independent samples, 2 independent experiments *two-tailed t-test*, n.s: not significant, horizontal line: mean). Source data

Next, we investigated whether inflammatory signalling is deregulated beyond the transcriptional level. Immunostaining analysis at H16 and D3 after treatment with 5-FU revealed that the levels of phosphorylated IRF3 (the active form of IRF3) were decreased in *Mda5*^−/−^ HSCs compared with WT HSCs at H16, but were similar at D3 (Fig. 6a and Extended Data Fig. 5a). The concentration of IFNβ, a type-I IFN that is induced directly by MDA5 signalling, was reduced in *Mda5*^−/−^ BM serum at D3, but not at H16 (Fig. 6b). Other cytokines, such as IL1a, IL23, IL10 and IL26, were also significantly increased in WT, but not in *Mda5*^−/−^, BM supernatant at D3, while few significant differences were observed at other time points (Extended Data Fig. 5b,c). The translocation of p65 to the nucleus was decreased in *Mda5*^−/−^ HSCs at H16 in comparison to the WT (Fig. 6c,d). Thus, inflammatory signalling is impaired in *Mda5*^−/−^ HSCs, potentially explaining their impaired activation after chemotherapy.

### MDA5 signalling regulates HSC activation in a cell-intrinsic manner

**Fig. 7:**
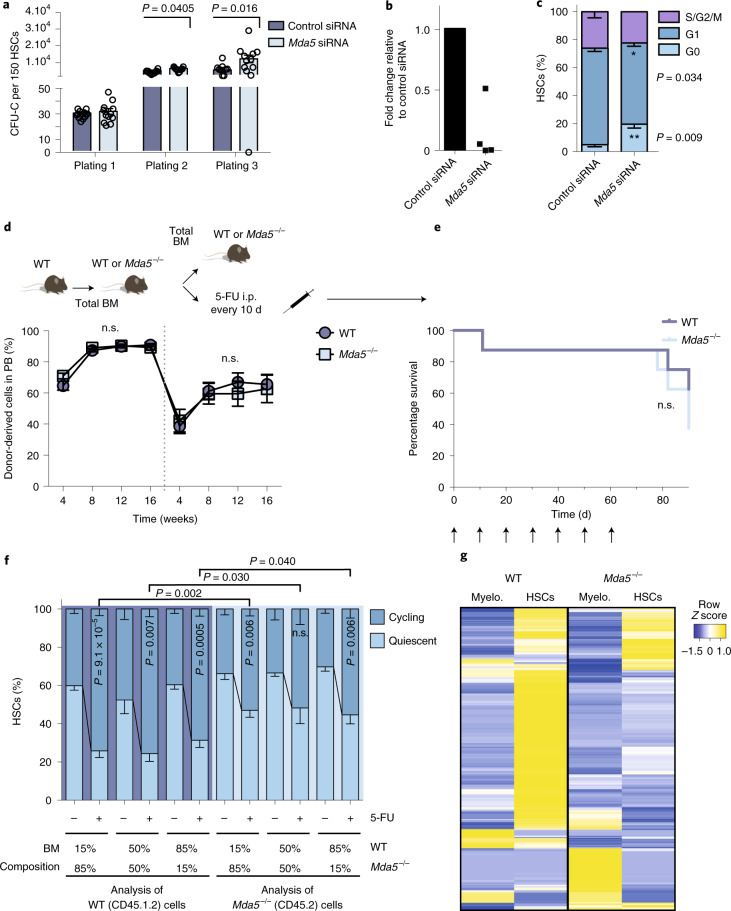
Intrinsic role of Mda5 in HSCs. **a**, Serial CFU-C assays in WT HSCs transfected with a control or an *Mda5* short interfering RNA (siRNA) pool. Colony counts were scored every 7 d. *n* = 12 technical replicates from *n* = 4 biologically independent experiments. Statistical analysis was performed using two-tailed *t*-tests. **b**, qPCR analysis of *Mda5* expression in WT HSCs transfected with a control or an *Mda5* siRNA pool. *n* = 4 biologically independent samples. **c**, Cell cycle analysis of HSCs transfected with a control (*n* = 4 biologically independent samples) or an *Mda5* siRNA pool (*n* = 7 biologically independent samples). Data are mean ± s.d. Statistical analysis was performed using two-tailed *t*-tests. **d**, The percentage of donor-derived cells in the peripheral blood of WT or *Mda5*^−/−^ primary recipients (week 4: *n* = 12 (WT) and *n* = 14 (*Mda5*^−/−^); and weeks 8, 12 and 16: *n* = 13 (WT and *Mda5*^−/−^) biologically independent samples) and secondary recipients (*n* = 8 (WT) and *n* = 7 (*Mda5*^*−/−*^) biologically independent samples). The dotted line separates the primary from secondary transplantation. Data are mean ± s.e.m. Statistical analysis was performed using two-tailed *t*-tests. **e**, Kaplan–Meier survival curve of WT or *Mda5*^−/−^ primary recipient mice after 5-FU injections every 10 d, 16 weeks after intravenous injection of total BM cells from WT mice. *n* = 8 mice. Statistical analysis was performed using the log-rank (Mantel–Cox) test; n.s., not significant. **f**, The cell cycle status of HSCs in chimaeras injected with the indicated ratios of WT and *Mda5*^−/−^ BM. Left, WT HSCs gated on CD45.1^+^CD45.2^+^ (CD45.1.2) cells. Right, *Mda5*^−/−^ HSCs gated on CD45.2^+^ cells, and the BM composition is indicated below. The groups were injected with 5-FU 4 d before the analysis. Data are mean ± s.d. No 5-FU: *n* = 4; with 5-FU: *n* = 6 (15:85), *n* = 5 (50:50), *n* = 9 (85:15) biologically independent samples in *n* = 2 independent experiments. Statistical analysis was performed using two-tailed *t*-tests. **g**, Heat map of the normalized fold change in the union of IRGs that are upregulated in WT HSCs and in WT myeloid (Myelo.) cells or *Mda5*^−/−^ HSCs and *Mda5*^−/−^ myeloid cells at H16 after 5-FU treatment compared with D0. Fold change cut-off = 1.5. *P*_adj_ < 0.05. Source data

As *Mda5* is knocked out in all tissues, we examined whether the phenotype of *Mda5*^−/−^ HSCs is cell intrinsic. Knockdown of *Mda5* in HSCs in vitro led to enhanced colony-forming-unit capacity, indicating that the function of MDA5 in HSCs is cell intrinsic (Fig. 7a–c). We next performed transplantation experiments of WT HSCs into *Mda5*^−/−^ or WT mice. We used these transplanted animals to perform the following secondary challenges: transplantation to secondary WT or *Mda5*^−/−^ recipients or repeated 5-FU injections every 10 d. Nevertheless, we found no significant differences between HSCs hosted into *Mda5*^−/−^ or WT mice (Fig. 7d,e). Next, we examined whether BM haematopoietic cells could contribute to the activation of HSCs. We performed transplantations with mixed WT and *Mda5*^−/−^ BM cells at different ratios (85:15, 50:50, 15:85) and, 2 months after transplantation, we challenged the mice with 5-FU and performed cell cycle analysis 4 d after. The 50:50 chimaeras showed that *Mda5*^−/−^ HSCs remain more quiescent than their WT counterparts (Fig. 7f). The ability of WT HSCs to exit quiescence after chemotherapy did not change even when 85% of the cotransplanted BM was *Mda5*^−/−^ (Fig. 7f). Furthermore, *Mda5*^−/−^ HSCs retained their quiescence even when 85% WT BM cells were co-transplanted (Fig. 7f).

We also investigated whether TE families and inflammatory signalling are upregulated at H16 after chemotherapy in myeloid cells (Mac1^+^Gr1^+^) from WT and *Mda5*^−/−^ mice. There was no upregulation of TE families in WT or *Mda5*^−/−^ myeloid cells in contrast to HSCs. Concomitantly, only 11 genes were upregulated and 21 were downregulated in WT myeloid cells, and 37 genes were upregulated and 92 downregulated in *Mda5*^−/−^ myeloid cells in comparison to hundreds of deregulated genes in HSCs (fold change cut-off = 1.5; *P*_adj_ < 0.05; Supplementary Tables 32–34). Few upregulated IRG genes were identified in WT or *Mda5*^−/−^ myeloid cells (Fig. 7g). These results show that the role of MDA5 in HSC activation is mostly cell intrinsic.

### Overexpression of TEs leads to HSC activation, whereas knockdown favours HSC quiescence

**Fig. 8:**
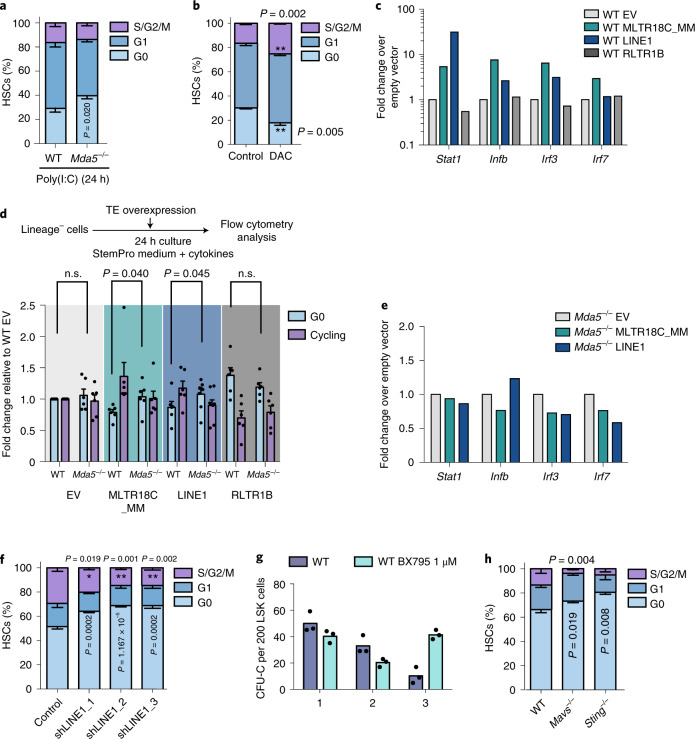
TE overexpression leads to HSC activation and knockdown leads to quiescence. **a**, Cell cycle analysis of WT or *Mda5*^−/−^ HSCs 24 h after poly(I:C) injection. *n* = 4 biologically independent samples. Data are mean ± s.d. Statistical analysis was performed using two-tailed *t*-tests. **b**, Cell cycle analysis of WT HSCs 72 h after decitabine (DAC) treatment or without DAC (control). *n* = 3 biologically independent samples. Data are mean ± s.d. Statistical analysis was performed using two-tailed *t*-tests. **c**, qPCR analysis of IRGs in WT HSCs transfected with an EV or different TE copies (both strands) as indicated. *n* = 2 biologically independent samples and experiments. **d**, The fold change relative to WT transfected with an EV of WT or *Mda5*^−/−^ HSCs in G0 or cycling after transfection with EV or the indicated TE copies (both strands). *n* = 6 biologically independent samples and experiments. Data are mean ± s.d. Statistical analysis was performed using two-tailed *t*-tests; n.s., not significant. **e**, qPCR analysis of IRGs in *Mda5*^−/−^ HSCs transfected with an EV or the indicated TE copies (both strands). *n* = 2 biologically independent samples and experiments. **f**, Cell cycle analysis of WT HSCs after transfection with control shRNA or knockdown of LINE1 with three different specific shRNAs. *n* = 3 biologically independent experiments with *n* = 2 replicates each. Statistical analysis was performed using two-tailed *t*-tests. Data are mean ± s.d. **g**, Serial CFU-C assay of BM HSCs from WT mice cultured for 48 h in the absence (WT) or presence of 1 μM TBK1 inhibitor (WT BX795). Colony counts were scored every 7 d. Representative of *n* = 2 independent experiments (*n* = 3 technical replicates). **h**, Cell cycle status of WT (*n* = 5 biologically independent samples) or *Mavs*^−/−^ (*n* = 11 biologically independent samples) or *Sting*^−/−^ (*n* = 3 biologically independent samples) HSCs determined by flow cytometry with Ki67 and Hoechst staining. *n* = 2 independent experiments. Data are mean ± s.d. Statistical analysis was performed using two-tailed *t*-tests. Source data

**Extended Data Fig. 6:**
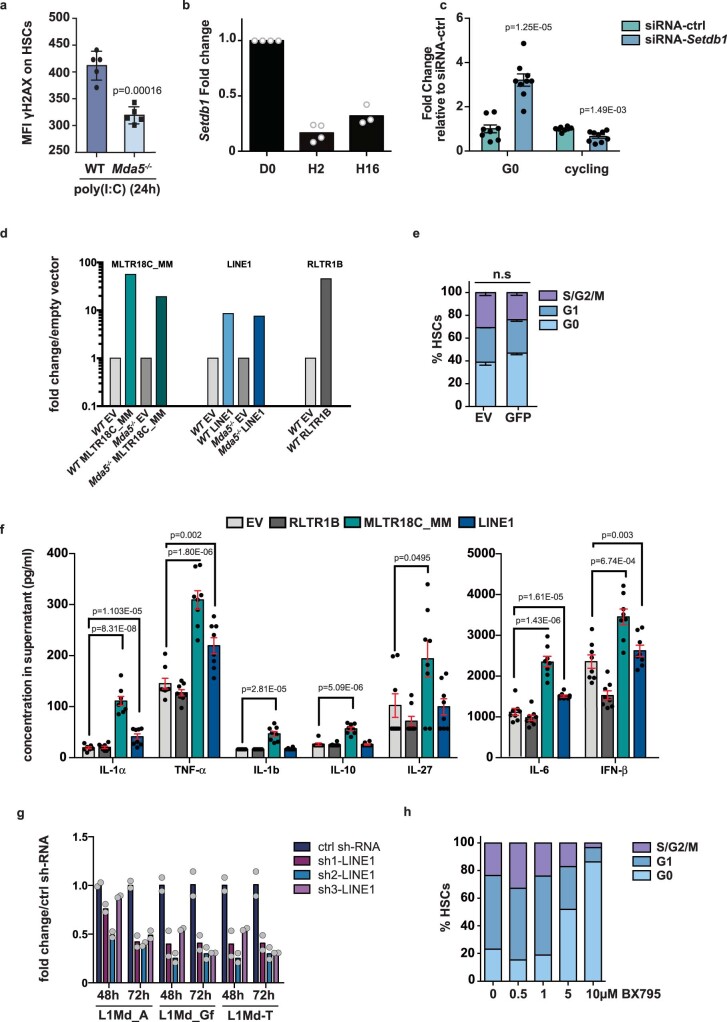
TE expression affects HSC cell cycle. **a**, Bar chart depicting the median fluorescence intensity (MFI) of γH2AX signal of WT or *Mda5*^*-/-*^ HSCs 24 h after poly(I:C) injection (n = 5 biologically independent samples, mean±s.d., *two-tailed t-test)*. **b**, RT-qPCR analysis of WT HSCs at D0, H2 and H16 after 5-FU injection for *Setdb1* (n = 4 biologically independent samples for D0 and H2, n = 3 for H16 biologically independent samples in two independent experiments). **c**, Cell cycle analysis of WT HSCs after transfection of control or *Setdb1* siRNA (n = 8 biologically independent samples for control siRNA and n = 9 for si*-Setdb1* in two independent experiments, *two-tailed t-test*, mean + /-s.d). **d**, qRT-PCR analysis of HSCs 20 h after transfection with empty vector (EV), or both strands of the indicated TE copies in WT or *Mda5*^*-/-*^ HSCs (n = 2 biologically independent samples). **e**, Cell cycle analysis of WT HSCs transfected with empty vector (EV) or the vector expressing GFP (n = 2 for EV and n = 3 for GFP biologically independent samples, *two-tailed t-test*, mean-s.d, n.s. non-significant). **f**, Measurement of secreted cytokines in supernatant of WT HSCs transfected as indicated (n = 8 biologically independent samples, 2 independent experiments, mean ± s.e.m, *two-tailed t-test*) **g**, qRT-PCR analysis of LSK cells 48 h or 72 h after knock-down of LINE1 families (n = 2 biologically independent samples). **h**, Cell cycle analysis of WT HSCs 48 hours after culture in presence of the indicated concentration of TBK1 inhibitor (BX795) (n = 2 biologically independent samples). Source data

As MDA5 is activated by double-stranded RNA ligands such as polyinosinic:polycytidylic acid (poly(I:C))^5,31^, we reasoned that poly(I:C) should be able to induce WT HSCs, but not *Mda5*^−/−^ HSCs, to exit quiescence. Indeed, 24 h after injection, a significant proportion of *Mda5*^−/−^ HSCs remained in G0 compared with WT HSCs, and a reduced accumulation of γH2AX foci was observed in *Mda5*^−/−^ HSCs (Fig. 8a and Extended Data Fig. 6a). We next verified that TE transcripts are indeed important for HSC activation by MDA5. Ex vivo decitabine treatment (which is known to cause TE transcriptional activation^29,33^) of HSCs for 72 h led HSCs to exit from the quiescent G0 state and enter cell cycle (Fig. 8b). Notably, we also examined the role of SETDB1, which is a known regulator of TE silencing^34,35^, in haematopoietic regeneration after chemotherapy. qPCR analysis of *Setdb1* showed that it was downregulated after treatment with 5-FU, but knockdown of *Setdb1* in cultured HSCs resulted in cell cycle stalling and only one upregulated TE family, in agreement with previous studies^36^ (Extended Data Fig. 6b,c and Supplementary Table 35). However, the expression results may understimate the upregulated TE families since we did not observe significant downregulation of *Setdb1* on the RNA-seq, as is sometimes the case in experiments using siRNAs.

We next reasoned that overexpression of TE copies should lead to HSC cycling and activation of inflammatory signalling. We overexpressed both strands of three different TE copies that were found to be transcriptionally upregulated in HSCs after 5-FU treatment in our bulk or single-cell RNA-seq data, namely, MLTR18C_MM, RLTR1B, and a fragment of LINE1. Using qPCR, we verified the overexpression of TE copies (Extended Data Fig. 6d). By overexpressing GFP as a control-coding gene, we observed no difference in the activation of WT HSCs (Extended Data Fig. 6e). Expression of MLTR18C_MM and LINE1 fragment but not RLTR1B in WT HSCs led to the activation of inflammation, as reflected by qPCR analysis of inflammatory genes in sorted and transfected HSCs, and the secretion of cytokines in the milieu (Fig. 8c and Extended Data Fig. 6f). In accordance, overexpression of MLTR18C_MM and LINE1, but not RLTR1B, was sufficient to decrease the percentage of G0 WT HSCs in comparison to HSCs transfected with an empty vector (EV) (Fig. 8d). On the contrary, overexpression of the same elements in *Mda5*^−/−^ HSCs did not significantly change the number of cells in G0 compared to EV-transfected cells nor did it induce upregulation of inflammatory genes in sorted and transfected *Mda5*^−/−^ HSCs (Fig. 8d,e). Knockdown of LINE1 with three different short-hairpin RNAs (shRNAs) that affect the expression of all recent LINE1 families, L1Md_A, L1Md_Gf and L1Md_T led to enhanced HSC quiescence as more HSCs remained in G0 (Fig. 8f and Extended Data Fig. 6g). Thus, it is possible that TEs have a functional role in haematopoietic regeneration through activation of MDA5.

### MAVS, TBK1 and STING are required for HSC activation

MDA5 is not the only sensor present in HSCs and therefore, in theory, other sensors or downstream proteins could also affect HSC activation after stress. We treated WT HSCs with an inhibitor of TBK1 kinase, which is downstream of MDA5, RIG-I and STING^9,37^. As inhibition of TBK1 has been reported to affect the spindle assembly during mitosis^38^, we titrated the TBK1 inhibitor BX795 and used a concentration that does not affect the cell cycle. TBK1 inhibition led to an increase in the number of CFU-C colonies after replating (Fig. 8g and Extended Data Fig. 6h). Furthermore, knockout of *Mavs*, which encodes a downstream adaptor protein, or *Sting*, which encodes a DNA-sensing-associated molecule, showed that a greater percentage of *Mavs*^*−/−*^ or *Sting*^−/−^ HSCs remain in G0 in comparison to WT HSCs (Fig. 8h). These results show that DNA-sensor signalling through STING and signal mediators downstream of MDA5 and RIG-I regulate HSC activation.

**Extended Data Fig. 7:**
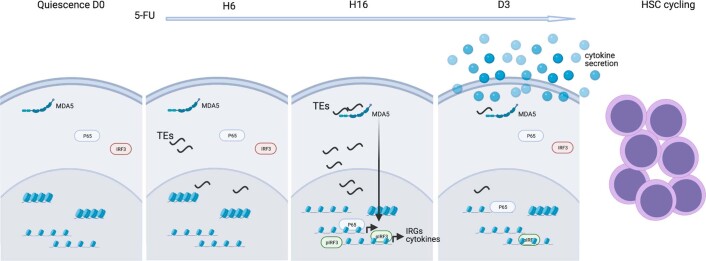
Effect of the TE-MDA5-Inflammation axis on HSC activation. Schematic showing that chromatin rearrangement occurs after chemotherapy concomitant to activation of TEs that are transcribed (H6-H16). TE transcripts bind to MDA5 to induce phosphorylation and thus activation of IRF3 and translocation of p65 to the nucleus (H16). This leads to activation of interferon responsive genes (H16) and secretion of proinflammatory cytokines (D3) followed by HSC cycling. Created with BioRender.com.

Collectively, our results show that chromatin accessibility changes after chemotherapy are followed by TE transcriptional upregulation. TEs activate the RNA sensor MDA5 to induce inflammatory signalling and HSC proliferation (Extended Data Fig. 7).

## Discussion

Our findings show that TEs, mainly ERVs and LINEs, are transcriptionally upregulated after chemotherapy and act as ligands for MDA5 to trigger an inflammatory response that results in HSCs exiting quiescence. Note that inverted *Alu* repeats were shown to be the primary ligands of MDA5 (refs. ^10,12^). Even though we did not identify such elements as deregulated after chemotherapy, it is possible that expression data of higher depth will reveal such deregulation. Moreover, further studies are needed to examine whether TEs expressed after chemotherapy are single-stranded, or double-stranded due to either bidirectional transcription or formation of double-stranded stretches.

Another outstanding question is the regulation of TE transcription after chemotherapy. We show that SETDB1—a well-known regulator of TE silencing^34,35,39^—is transcriptionally downregulated after chemotherapy but its knockdown in cultured HSCs did not lead to HSC activation. However, the role of SETDB1 may be different in chemotherapeutic stress in comparison to culture. Inflammatory signalling also regulates TE transcription^40,41^, and co-evolution of TEs and immune genes has been established^42^ in agreement with the observed differences in TE deregulation between WT and *Mda5*^−/−^ HSCs. Our findings are consistent with previous research showing that TEs are upregulated in HSCs after irradiation. However, in that study, a thrombopoietin-induced IFN-like response was shown to restrain LINE1 activity^21^. Our data indicate a second wave of IFN gene expression at D10 that could restrain TE transcription. Finally, phenomena such as pervasive transcription^43^ may influence TE transcription and need further delineation. It will also be interesting to examine whether editing by ADAR or RNA methylation occurs in TEs after chemotherapy as both mechanisms have been shown to prevent MDA5 activation^11,44,45^.

As inflammatory signalling is central to HSC activation, it is conceivable that other RIG-I-like receptors, particularly RIG-I, DNA-sensing pathways such as cGAS-STING or inflammasome components could have similar roles in haematopoietic regeneration. Indeed, it has been shown that the RIG-I is more abundant in multipotent haematopoietic progenitors versus myeloid cells^46^ and STING activation leads to HSC mobilization^47^, while NLRP3 has a role in HSC emergence^48^. The activation of DNA-sensing pathways was recently shown to be caused by R-loops in HSC development^49^. Our data suggest a role for different sensors in HSC activation. However, the role of MDA5 in haematopoietic regeneration may also depend on other functions besides its role in inducing inflammation, as activation of MDA5 has been associated with endoplasmic reticulum stress^50^, metabolism^51^ and autophagy^52^.

We have previously shown that an interplay between TEs and RIG-I-like receptors enhances HSC formation in a non-stress developmental setting^53^. Our data suggest that this TE–MDA5 coupled mechanism may also function under homeostatic conditions in the BM, as HSCs lacking MDA5 are more resistant to activation under homeostasis. We propose that the TE–MDA5 signalling axis buffers mild homeostatic or robust stress signals by titrating inflammatory signals that modulate HSC activation. Several other stress signals, including ageing^20^ and heat shock stress^17^ also activate TE expression. Thus, it is plausible that TE sensing by RNA/DNA sensors is a phenomenon that is used by diverse cell populations to respond to challenges.

## Methods

### Cell lines

HEK293T and OP9 cells were maintained at 37 °C and 5% CO_2_ and cultured in DMEM or alphaMEM containing glutamine (Gibco) respectively, supplemented with 10% fetal bovine serum and 1% penicillin–streptomycin.

### Mice

All mouse experiments were carried out in accordance with the guidelines of the Federation of European Laboratory Animal Science Association and following legal approval of the Regierungspräsidium Freiburg (35/9185.81/G-15/100, 35-9185.81/G-18/41, 35-9185.81/G-18/127, 35-9185.81/G-20/127). *Mda5*^−/−^ mice (B6.Cg-*Ifih1*^*tm1.1Cln*^/J)^31^ were purchased from the Jackson Laboratory and backcrossed into C57BL/6J WT mice (CD45.2^+^/Ly5.2). *Sting*^−/−^ mice bones (B6(Cg)-*Sting1*^*tm1.2Camb/J*^)^54^ and *Mavs*^*−/−*^ mice bones (*Mavs*^*tm1Tsc*^)^55^ and their respective controls were a gift from J. Rehwinkel. All of the animals were maintained at the animal facility of the Max Planck Institute of Immunobiology and Epigenetics under specific-pathogen-free conditions in individually ventilated cages with a light–dark cycle of 12 h–12 h at 20–24 °C under 45–65% humidity. For all genotypes, gender-matched female or male mice (aged 6 to 12 weeks), or bones, were used in the experiments.

### Antibodies, except for the LINE1-knockdown experiment

The following antibodies were purchased from BioLegend and used at a 1:400 dilution unless indicated otherwise: anti-CD45.1/Ly5.1 (APC-Cy7, A20), anti-CD45.2/Ly5.2 (Alexa Fluor 700, 104), anti-CD3e (FITC, 145-2C11), anti-CD11b/Mac-1 (1:1,600, FITC or PerCP-Cy5.5, M1/70), anti-Ly6C/Ly6G (1:1,600, FITC or PerCP-Cy5.5, RB6-8C5), anti-CD45R/B220 (FITC or APC, RA3-6B2), anti-Ter119 (FITC, Ter-119), anti-CD117/c-kit (Brilliant Violet 421 (1:600) or PE; BioLegend, or APC-H7, 2B8, (1:200) BD Bioscience), anti-Sca-1 (Pe-Cy7, E13-161.7), anti-CD48 (1:800, PerCP-Cy5.5, HM48-1), anti-CD150 (1:600, PE-Dazzle or 1:600 Brilliant Violet 605, TC15-12F12.2), anti-CD135/Flk2 (1:200, PE, A2F10.1, BD Pharmingen), anti-CD34 (1:30, Alexa Fluor 700 RAM34, eBioscience), anti-Ki67 (1:200, Alexa Fluor 647, 11F6), anti-CD201 (1:200, EPCR, PE anti-mouse, RCR16), anti-p-IRF3 (1:25, S396, D601M, rabbit monoclonal antibody 29047, Cell Signaling), goat anti-rabbit secondary (1:500, Alexa Fluor 647, A21245, Invitrogen), anti-γH2AX (1:100, Alexa Fluor 647 (Ser 139), 2F3) and anti-p65 (1:100, Alexa Fluor 488, p65, Santa Cruz Biotechnologies) antibodies.

### Sorting strategy

Throughout the text, HSCs refer to LSK/SLAM cells (Lin^−^Sca1^+^cKit^+^CD150^+^CD48^−^) or EPCR/SLAM unless indicated otherwise. LT-HSCs: LSKCD34^−^CD135^−^CD150^+^CD48^−^; MPP1: LSKCD34^+^CD135^−^CD150^+^CD48^−^; MPP2: LSKCD34^+^CD135^−^CD150^+^CD48^+^; MPP3: LSKCD34^+^CD135^−^CD150^−^CD48^+^; MPP4: LSK CD34^+^CD135^+^CD150^−^CD48^+^.

### HSC quantification and sorting by flow cytometry

For HSC quantification, tibiae, femurs and hip bones were crushed in staining buffer (PBS, 2% FBS, 1 mM EDTA). Erythroid cells were lysed in an ammonium-chloride-potassium buffer (150 mM NH_4_Cl, 10 mM KHCO_3_, 0.1 mM EDTA) for 5 min at room temperature. Cells were washed, resuspended in staining buffer and counted using a Casy Cell counter. For sorting, samples were enriched for HSCs by lineage depletion using a biotin-conjugated lineage cocktail (CD3e, CD11b/Mac-1, CD45R/B220, Ly-6/Ly6C, TER-119) for 20 min at 4 °C. Streptavidin nanobeads (MojoSort, BioLegend) were added for 20 min at 4 °C followed by magnetic separation for 4 min at room temperature. Then, 10^7^ cells per ml were stained with antibodies against CD117/c-kit, Sca-1, CD48, CD150 and, if indicated, CD135/Flk2, CD34 or EPCR, CD48 and CD150 for 20 min at 4 °C (for CD34, 90 min at 4 °C). Cells were washed, resuspended in 500 µl of staining buffer, and data were either acquired on a Fortessa FACS analyser or sorted using a FACS ARIAIII or FACS ARIAFusion (BD Biosciences). CD11b/Mac-1 was excluded after 5^−^FU or poly(I:C) injections. All data were analysed using FlowJo v.10.6.1.

### HSC culture

HSCs or lineage-negative cells were cultured in StemPro-34 medium with 2.5% StemPro-34 Supplement, (106439011, Gibco), 50 ng ml^−1^ mSCF, 25 ng ml^−1^ mTPO, 30 ng ml^−1^ mFlt3L, 1% penicillin–streptomycin and 2 mM l-glutamine.

### Transplantation experiments

WT mice and *Mda5*^−/−^ mice (CD45.2^+^/Ly5.2) were used as donors, WT mice (CD45.1^+^/Ly5.1) were used as competitors and WT mice (CD45.1^+^/CD45.2^+^) were used as recipients. No difference in engraftment was observed between CD45.1 and CD45.2 mice. When *Mda5*^−/−^ mice were used as recipients, WT mice (CD45.1^+^/CD45.2^+^) were used as donors and WT mice (CD45.1^+^/Ly5.1) were used as competitors. Transplantations were conducted at a 1:1 ratio of donor and competitor HSCs (LSK/SLAM). For primary transplantation, we estimated the number of HSCs in the BM and intravenously injected BM corresponding to 250 donor and competitor HSCs into lethally irradiated (9.5 Gy) recipients. For secondary transplantations, 3 × 10^6^ total BM cells from primary recipients with similar chimerism were transplanted into lethally irradiated (9.5 Gy) recipients. Peripheral blood chimerism was checked every 4 weeks for 16 weeks. For the mixed chimaera experiments, 30 × 10^6^ total BM cells from WT (CD45.1^+^/CD45.2^+^) and *Mda5*^−/−^ (CD45.2^+^/Ly5.2) mice at different ratios were intravenously injected into lethally irradiated (9.5 Gy) WT (CD45.1^+^/Ly5.1) recipients. Then, 8 weeks later, recipients were injected intraperitoneally with 150 mg kg^−1^ body mass 5-FU (Sigma-Aldrich, F6627) or PBS and BM cells were analysed after 4 d.

For HSC transplantation after culture, 200 HSCs (LSK/SLAM) were cultured for 48 h and each well was co-injected with 200,000 WT (CD45.1^+^/Ly5.1) BM cells into one recipient.

For engraftment, 20 µl of blood was obtained from the tail vein. Erythrocytes were lysed and after washing, samples were resuspended in 100 µl of staining solution for 20 min at 4 °C with anti-CD45.1/Ly5.1, anti-CD45.2/Ly5.2, anti-CD3e, anti-CD11b/Mac-1, anti-Ly6C/Ly6G, anti-CD45R/B220 and anti-Ter119 antibodies.

### Homing assay

Mice were euthanized 16 h after transplantation, and the presence of donor cells (LSK) in the BM was addressed by flow cytometry using antibodies against CD45.1/Ly5.1, CD45.2/Ly5.2, CD117/c-kit, Sca-1, CD3e, CD11b/Mac-1, CD45R/B220, Ly-6/Ly6C and TER-119, and a cocktail of FITC lineage antibodies.

### Side population staining

In brief^56^, 10^7^ BM cells per ml were resuspended in DMEM with penicillin–streptomycin, 10 mM HEPES and 2% FBS. Hoechst 33342 (B2261, Sigma-Aldrich) was added at 5 µg ml^−1^ for 90 min at 37 °C. After washing, cells were stained with anti-CD117/c-kit, anti-Sca-1 and anti-CD150 antibodies in staining buffer at 4 °C for 20 min. Cells were resuspended in cold HBSS (14170-112, Gibco Invitrogen) with 10 mM HEPES and 2% FBS. Propidium iodide (2 µg ml^−1^; P-4170, Sigma-Aldrich) was added before analysis.

### siRNA knockdown of *Mda5*

Lineage-negative cells from WT or *Mda5*^−/−^ mice that were cultured as described above were transfected with DharmaFECT1 (T-2001-02, Dharmacon) with 50 nM control non-targeting or *Mda5* or *Setdb1* siRNA (D-001810-10-05, L-065328-00-0005, L-040815-01-0005, Dharmacon) together with siGLO (D-001630-01-05). After 48 h, cells were collected and stained for LSK/SLAM for 30 min at 4 °C in the dark. HSCs were fixed for cell cycle and sorted HSCs were used for CFU-C assays and RT–qPCR. In the case of *Setdb1* knockdown, HSCs (LSK/SLAM) were sorted and processed for RNA-seq or cell cycle analysis.

### CFU-C assays

LSK/SLAM HSCs (200) were sorted in a 96-well plate, cultured and, when indicated, incubated for 48 h with 1 µM BX795 (TBK1 inhibitor, 4318, Tocris). Each well was transferred into 900 μl of Mouse Methylcellulose Complete Media (HSC007, R&D systems), and split into 3 separate wells of a 24-well plate. Colonies were counted after 7 d at 37 °C and 5% CO_2_. For replatings, cells were washed with PBS and analysed by flow cytometry to estimate the number of LSK cells. LSK cells (200) were replated in fresh methylcellulose and counted after 7 d. For knockdown experiments, 150 HSCs were plated and counted after 7 d. Then, 10,000 cells per well were replated in fresh methylcellulose, and colonies were counted after 7 d.

### 5-FU, cytarabine, cyclophosphahmide and poly(I:C) treatment

5-FU, cytarabine and poly(I:C) were injected intraperitoneally at 150 mg kg^−1^ (Sigma-Aldrich, F6627), 100 mg kg^−1^ (PHR1787, Supelco) and 10 µg g^−1^ (P9582, Sigma-Aldrich), respectively. Cyclophosphamide was injected intravenously at 200 mg kg^−1^ (C0768, Sigma-Aldrich).

### Decitabine treatment of HSCs

Lineage-negative cells were isolated and cultured as described above with the addition of 1 μM decitabine (A3656, Sigma-Aldrich) for 72 h. Cells were fixed for cell cycle analysis.

### Mitochondrial mass and ROS quantification

BM cells were stained in StemPro 34SFM medium with MitoTracker Green (M7514, Thermo Fisher Scientific) at 50 nM for 15 min at 37 °C or with CellRox Deep Red reagent (C10422, Thermo Fisher Scientific) at 500 nM for 30 min at 37 °C. HSCs (LSK/SLAM) were stained, washed and analysed.

### Cell cycle staining and phosphorylated IRF3 staining

HSCs (LSK/SLAM) were stained, sorted, washed and resuspended in fixed intracellular Fixation Buffer (00-8222-49, Thermo Fisher Scientific) for 10 min at 4 °C. Cells were washed and resuspended in permeabilization buffer (00-8333-56, Thermo Fisher Scientific) with anti-Ki67 antibodies and Hoechst 33258 (H3569, Life technologies) for 30 min at 4 °C or with anti-p-IRF3 antibodies overnight at 4 °C. For p-IRF3 staining, cells were washed with permeabilization buffer and stained with AlexaFluor647 (A21245, Invitrogen) for 30 min at room temperature. Cells were washed with permeabilization buffer and analysed. The Zombie Fixable Viability Kit (423105, BioLegend) was used for dead cell exclusion in cell cycle staining.

### γH2AX and p65 staining

HSCs (LSK/SLAM; 1,000 per slide) were sorted onto PolyPrep l-lysine-coated (Sigma-Aldrich) slides (Ibidi µ-slide) and fixed with intracellular fixation buffer (00-8222-49, Thermo fisher Scientific) for 10 min at 4 °C. Cells were washed and resuspended in permeabilization solution (00-8333-56, Thermo Fisher Scientific) containing 0.1% BSA for 30 min at room temperature. Cells were stained overnight at 4 °C with γH2AX or p65 antibody. After washing, 1 µg ml^−1^ of Hoechst 33258 was added. Images were acquired using LSM880 (Zeiss), airyscan processed and analysed using Imaris v.9.2 spot detection algorithm (Bitplane). For p65 staining, the Hoechst channel image was used to manually detect individual nuclei. For each nucleus, we measured the integrated p65 intensity inside the nucleus. A region of interest (ROI) corresponding to the entire cell was then obtained and the integrated p65 intensity inside the ROI was measured. The cytoplasmic integrated p65 intensity was obtained by subtracting the integrated p65 intensity inside the nucleus from the integrated p65 intensity inside the ROI. Alternatively, the pixel intensity (grey value) of p65 and Hoechst was measured along a line in the overlay plots and displayed as histograms.

### Cytokine quantification

The LEGENDplex Mouse Inflammation Panel (BioLegend) was used according to the manufacturer’s instructions. In brief, after bone crushing, the cell suspension was centrifuged at 1,500 r.p.m. for 5 min at 4 °C. The BM serum was stored at −80 °C. Samples were diluted 1:1 and incubated with beads conjugated with the respective antibodies. For Extended Data Fig. 6d, 700 µl of supernatant was collected after 24 h of culture, cells and debris were excluded by centrifugation and samples were processed as described above.

### Cell division assays

HSCs (LSK/SLAMCD135^−^) were single sorted in Terazaki microtest plates (654102, Greiner) in the medium described above. Then, 1 h after sorting, the presence of cells was verified and, 24 h and 48 h later, the number of cells per well was counted using Axio Vert.A1 (Zeiss).

### TE overexpression experiments

MLTR18C_MM, RLTR1B and a part of LINE1 were synthesized by and cloned in pCCAGGs-IRES-Puro (gift from the Jenuwein laboratory). Clones containing the sense or antisense sequences were verified by sequencing. Lineage^−^ cells (4 × 10^6^) were electroporated with 2 µg of sense and 2 µg of antisense constructs using P3 Primary Cell 4D-NucleofectorTM XKitL (V4XP-3024, Lonza). pCCAGGs-IRES-Puro was used as an EV and pmaxGFP plasmid as transfection efficiency control. Then, 24 h after electroporation, HSCs (LSK/SLAM) were stained and fixed for cell cycle analysis or sorted and total RNA was isolated (D4013, Zymo) and reverse-transcribed using SuperScript III (18080-051, Invitrogen) or PrimeScript RT (RR047A, Takara). RT–qPCR reactions were performed using the TB Green Premix (RR42LR, Takara) in a StepOnePlus Real-Time PCR machine (Applied Biosystems). Expression was quantified over EV and normalized to the expression of HPRT or beta actin.

### LINE-1 knockdown

BM was extracted from femur, pelvic bone, tibias and spine by crushing. c-Kit^+^ cells were isolated using magnetic anti-CD117 microbeads (130-091-224, Miltenyi Biotec) and an autoMACs magnetic cell-separator. For HSC (LSK/SLAM) isolation, cells were stained using an anti-lineage BV605 antibody cocktail (1:400) and antibodies against Sca-1 PerCPCy5.5 (122523, E13-161.7clone, BioLegend), c-Kit APCe780 (47-1171-82, clone 2B8, eBioscience), CD48-Alexa Fluor700 (56-0481-82, clone HM48-1, eBioscience) and CD150-PE-Cy7 (115913, clone TC15-12F12.2, BioLegend) (1:200 for the rest of the antibodies). DAPI was used for dead cell exclusion. Cell were sorted using the FACSAriaIII system. shRNAs were designed as previously described^57,58^. Vesicular stomatitis virus glycoprotein–pseudotyped lentivirus was prepared using a four-plasmid system (Transfer vector-, Gag/Pol-, Rev/Tat-, and envelope plasmid) by cotransfection of HEK293T cells using TransIT293 (Mirus)^58^. Supernatant was collected 48 h later, cleared, titred onto HEK293T cells and stored at −80 °C. LSK cells from mice (aged 6–10 weeks) were transduced with lentivirus as described previously^58^. Non-tissue-culture 96-well plates were coated with Retronectin (TaKaRA Bio), and lentiviral particles (multiplicity of infection of 25) were spinoculated for 1 h at 1,000*g* at room temperature. Wells were washed with PBS, and 15,000 freshly isolated LSK cells were resuspended in 200 µl StemSpan (09600, StemCell Technologies) with recombinant mouse SCF (10 ng ml^−1^), TPO (20 ng ml^−1^), IGF2 (20 ng ml^−1^) (PeproTech), 10 ng ml^−1^ recombinant human FGF1 (R&D Systems) and 5 µg ml^−1^ protamine sulfate (Sigma-Aldrich). Then, 48 h after transduction, the medium was slowly removed, and the cells were washed and resuspended in PBS + 1.5% FBS. For cell cycle analysis, LSK cells transduced with control or L1 shRNAs and cultured for 5 d, at which point mCherry^+^ HSC (LSK/SLAM) cell cycle was examined by flow cytometry using Ki67. Total RNA was isolated (74004, Qiagen) and reverse-transcribed (4368814, Invitrogen). To confirm that HSCs express full-length L1, purified mRNA was reversed transcribed using a sense-strand L1-specific primer recognizing the 3′ end of *ORF2*, as described previously^59^. qPCR was performed using the Fast SYBR Green Master Mix (Applied Biosystems) on an ABI StepOnePlus thermal cycler (Applied Biosystems). Gene knockdown efficiency in LSK cells was quantified using RT–qPCR.

### Oligos and primers

A list of oligos and primers is provided in the last sheet of Supplementary Table 36.

### RNA-seq

For RNA-seq (2–3 biological replicates per sample, 2,000cells per sample), HSCs (LSK/SLAM) from WT and *Mda5*^−/−^ mice were sorted and RNA was isolated using either the PicoPURE Arcturus kit (KIT0204, Applied Biosystems) for HSCs or the RNeasy Mini Kit (74104, Qiagen) for myeloid cells. For HSCs, cDNA libraries were prepared using SMARTseqv4 (R400752, Takara) with 12 cycles of amplification. The NEBNext Ultra II FS DNA kit (E7805S) was used to generate barcoded sequencing libraries. cDNA library (3–10 ng) was fragmented for 22.5 min, adapters were ligated and libraries were amplified using cycle numbers according to input material. The NEB Next low input library kit (E6420) was used for the preparation of RNA-seq libraries from myeloid cells.

### RNA-seq analysis of genes

Paired-end 101 bp reads for WT and *Mda5*^−/−^ samples were generated using the Illumina Hiseq 3000 or NovaSeq 6000 system. Adapter sequences were trimmed using Trimmomatic (v.0.36)^60^ and then reads were aligned to mouse genome version GRCm38/mm10 using STAR aligner (v.2.5.3a)^26^. Samtools (v.0.1.19)^61^ was used for data filtering and file format conversion, while the HTseq count (v.0.5.4p3)^62^ algorithm was used to assign aligned reads to exons using the following command line: «htseq-count --s no --m intersection -nonempty». Differentially expressed genes were identified using the DESeq R package^63^, and genes with fold change cut-off of 1.5 and *P*_adj_ < 0.05 were considered to be differentially expressed (DEGs). All times points were normalized together. Heat maps showing the normalized fold-change of deregulated genes were made in R using the gplots package (https://cran.r-project.org/package=gplots) and heatmap.2 function. Stack bar graphs representing the number of DEGs were constructed using the R package Shiny (https://shiny.rstudio.com/).

### RNA-seq analysis of TE families

Analysis was performed as described previously^64^. Paired-end 101 bp reads for WT and *Mda5*^−/−^ samples were generated using the Illumina Hiseq 3000 or NovaSeq 6000 system. Adapter sequences were trimmed with Trimmomatic (v.0.36)^60^ and then reads were aligned to GRCm38/mm10 using STAR aligner (v.2.5.3a)^26^ using the following options: -readFilesCommand -outFilterMultimapNmax 100 -winAnchorMultimapNmax 100 -outMultimapperOrder Random -outSAMmultNmax 1 -outSAMtype BAM -outFilterTypeBySJou -alignSJDBoverhangMin 1 -outFilterMismatchNmax. Using the multimapped aligned files, TEtranscript (v.2.0.3)^27^ was used with the option --mode multi to estimate TE abundances. Annotation files were constructed from RepeatMasker (http://www.repeatmasker.org). Differentially expressed TE families were identified using the DESeq R package^63^, whereby TE families were normalized together with the genes. TE families with a fold-change cut-off of 1.5 and *P*_adj_ < 0.05 were considered to be differentially expressed. Heat maps showing expression of deregulated TE families were made in R using the pheatmap package^65^.

### RNA-seq analysis of TE copies

Multimapped reads were filtered with Samtools MAPQ > 50 to extract the uniquely mapped reads. The HT-seq count (v.0.5.4p3.) algorithm^62^ was applied to assign aligned reads to the genomic instances of TE copies using the following command line ‘htseq-count --s no --m intersection --nonempty’. Annotation files were constructed from RepeatMasker (http://www.repeatmasker.org). Differentially expressed TE copies were identified with the use of the DESeq R package^63^ (normalized pairwise to achieve good clustering), and TE copies with fold change cut-off of 1.5 and *P*_adj_ < 0.05 were considered to be differentially expressed. Heat maps showing expression of deregulated TE copies were made in R using the gplots package (https://cran.r-project.org/package=gplots) and heatmap.2 function.

#### TE copy gene proximity

The coordinates of the genomic regions of the upregulated TE copies were extracted from UCSC (https://genome.ucsc.edu/) and assigned to the closest gene using the closestBed subcommand of BEDtools --b mm10_tss.bed and the following parameters: -D ‘a’ -t ‘first’. By taking a window of 30 kb upstream and downstream from the TSS of the genes, we created a list of genes that were proximal to upregulated TE copies and selected the genes that were significantly deregulated in our RNA-seq analysis. Significance of the overlap was evaluated using the Fisher’s and hypergeometric tests. The expected background was determined by randomly sampling an equal number of the remaining genes and determining the number of genes located within 30 kb of the deregulated TE copies that were identified as proximal to deregulated genes. The parameter --exl was used to exclude gap files, blacklist regions, chrM and restrict the randomization within the corresponding genomic regions defined above. Sampling was repeated 10,000 times and the mean number of overlaps from all of the shuffled datasets was used to determine the expected counts of overlaps with a binomial test, which was performed in R using the binom.test function.

### ATAC-seq

For ATAC-seq (2 biological replicates per sample, ~5,000 cells per sample), HSCs (LSK/SLAM) were sorted and library preparation was performed exactly as described previously^66^ using the Nextera DNA Library Prep kit (15028212, Illumina).

### ATAC-seq analysis

ATAC-seq paired-end 75 bp reads were generated using the Illumina Hiseq 3000 system. Adapter sequences were trimmed using Trimmomatic (v.0.36) and TrimGalore (v.0.4.3). Bowtie2 (v.2.1.0)^67^ using the «--very-sensitive» parameter was used for aligning ATAC-seq reads to GRCm38/mm10 and Samtools (v.0.1.19)^61^ for data filtering and file format conversion. Duplicate reads, blacklist regions and chrM were removed before peak calling. All filtered .bam files were converted to bedgraphs using the deepTools bamCoverage subcommand, with the reads per kilobase of transcript, per million mapped reads (RPKM) normalization method. MACS2 (v.2.1.0) algorithm^68^ was used for peak identification (*P*-value cut-off = 1 × 10^−8^). Gained peaks for each time point, compared to D0 were identified from the narrow peaks in two steps. First, the peak lists from two times points (for all time points compared to D0) were merged with the subcommands cat and mergeBed to obtain consensus peaks. Second, for each time point and with intersectBed and the parameters --a <consensus peaks> --b <narrow peaks> --wa --u and subsequently --v, we compare and subtract them, to take the gained and lost peaks for each time point compared to D0. The reads of these consensus peaks were counted and a statistical model based on edgeR^69^ was used to identify the significantly differential peaks. To identify the common peaks between the time points, DESeq which uses the Negative Binomial distribution to compute a *P* value and a fold change for each estimated peak was used. Peaks highly enriched in comparison to the rest were considered gained. Annotation of peaks to genes (100 kb upstream and 25 kb downstream from the TSS) and genomic distribution of accessible regions identified by MACS2 was performed using BEDTools and the -closetBed and -intersectBed subcommands, respectively. Clustering of regions was generated with the ComputeMatrix function of DeepTools^70^, using the reference point --referencePoint center -b 3000 -a 3000 -R <bed files> -S <bigwig files> as parameters, except for the WT versus *Mda5*^−/−^ comparison, for which we used the following pararameters as the reference-point: --referencePoint center -b 2500 -a 2500. The function plotHeatmap from the same package was used for displaying the average profiles heat map.

### Digital genomic footprinting for ATAC-Seq

The produced .bam files from ATAC-seq data were merged using samtools merge (v.1.3.1)^61^. Digital genomic footprinting was performed using dnase_footprints of the Wellington pyDNase package (v.0.2.4)^71^ on total merged ATAC peaks with a *P*-value cut-off of 1 × 10^−5^, using -A as a parameter to enable ATAC mode, resulting in a coordinate shift 5′ and 3′ by +4 bp and −5 bp, respectively. Motif overrepresentation and average profile analyses were performed using dnase_average_profile.py of the Wellington pyDNase package^71^, on WT-only and WT versus *Mda5*^−/−^ footprints. For calculating the footprinting occupancy scores, a Python script from Wellington pyDNase package (wellington_score_heatmap.py) was used. First, footprinting occupancy scores were log_2_-transformed and then two-tailed paired *t*-tests were performed in R, as if each value corresponds to the same chromosomal region, and not assuming any direction in the relationship between both samples.

### ATAC-seq analysis of TEs

Adapter sequences were trimmed using Trimmomatic v.0.36 and TrimGalore v.0.4.3. Reads were mapped to GRCm38/mm10 using the Bowtie2 algorithm (v.2.1.0)^67^, using the «--very-sensitive» parameter enabling multiple mapping. Samtools^61^ filtering MAPQ > 10 was performed and all .bam files were converted to bedgraphs using the deepTools bamCoverage subcommand, dividing them into bins of size 50 and using the RPKM normalization method. Reads mapping to mitochondrial DNA or black-list regions were discarded. Significantly enriched peaks were detected using the MACS2 (v.2.1.0) algorithm^68^ with a *P*-value cut-off of 1 × 10^−5^. Peaks with a size OF smaller than 300 bp were discarded. Gained peaks for each time point, compared to D0, were identified as described above. Annotation files were constructed from RepeatMasker. Differentially accessible open chromatin regions were scanned for enriched short-sequence motifs using HOMER software59 with the ‘findMotifsGenome.pl’ command. To determine the enrichment (or depletion) of ATAC peaks nearby each TE family in relation to the genomic abundance of such families (compared to a random shuffling of such TEs), we used the Perl script TE-analysis_Shuffle_bed.pl from the software TEanalysis^72,73^, (https://github.com/4ureliek/TEanalysis) with the following parameters: -l none -n 1000 -o 10 along with the TE bed file +1 kb upstream the beginning of each TE and 1 kb downstream the end of each TE. The significance of enrichment was estimated using binomial and hypergeometric tests. Subsequently, we evaluated the expression of each of the TE families within significantly enriched ATAC peaks.

Clustering of regions was generated using the ComputeMatrix function of DeepTools^70^ using the reference point --referencePoint center -b 2500 -a 2500 -R <bed files> -S <bigwig files> as parameters. The function plotHeatmap from the same package was used for displaying the average profiles heat map.

### GO analysis and upstream regulator analysis

GO and pathway analysis was performed using Metascape (https://metascape.org/)^74^ (*P* ≤ 0.05). Upstream regulator analysis was performed through the use of IPA (QIAGEN Inc., https://www.qiagenbioinformatics.com/products/ingenuitypathway-analysis).

### Single-cell RNA-seq

Sorted HSCs (LSK/SLAM-cell numbers are shown in the figure legends) cells from two male mice per genotype and per condition were used. Single-cell RNA-seq was performed using the mCEL-Seq2 protocol, an automated and miniaturized version of CEL-Seq2 on a mosquito nanolitre-scale liquid-handling robot (TTP LabTech)^75,76^. Fifty-six libraries with 96 cells each were sequenced using the Illumina HiSeq 3000 or NovaSeq 6000 sequencing system (pair-end multiplexing run) at a depth of ~130,000–200,000 reads per cell. For quantification of transcript abundance, paired-end reads were aligned to the transcriptome using bwa (v.0.6.2-r126) using the default parameters^77^. The transcriptome contained all gene models on the basis of the mouse ENCODE VM9 (UCSC) comprising 57,207 isoforms, with 57,114 isoforms mapping to fully annotated chromosomes (1 to 19, X, Y, M). All isoforms of the same gene were merged to a single gene locus. Gene loci overlapping by >75% were merged to larger gene groups, resulting in 34,111 gene groups. The right mate of each read pair was mapped to the ensemble of all gene loci and to the set of 92 ERCC spike-ins in the sense direction^78^. Reads mapping to multiple loci were discarded. The left read contains the barcode information: first six bases, unique molecular identifier (UMI); second six bases, cell-specific barcode; remainder read, polyT stretch. For each cell barcode, the number of UMIs per transcript was counted and aggregated across all transcripts derived from the same gene locus. On the basis of binomial statistics, the number of observed UMIs was converted into transcript counts^79^. For the quantification of TEs, to tag the reads according to UMI and cell barcode information, fastq files were processed using umitools v.0.5.1 with the flags bc-pattern=NNNNNNCCCCCC and filter-cell-barcode. The tagged fastq files were then mapped to the GRCm38 genome assembly using STAR v.2.5.3a with the following parameters --runThread 4 --readFilesCommand zcat --outFilterMultimapNmax 100 --winAnchorMulitmapNmax 100 --outSAMtype BAM SortedByCoordinate. The reads from the .bam files were then assigned to TEs using featureCounts from the subread-1.5.3 package together with the following parameters: --t exon --g gene_id --T 4 --R BAM and by using a .gtf file with annotated transposable elements (http://hammelllab.labsites.cshl.edu/software/#TEtranscripts). The annotated data were sorted and indexed using Samtools v.1.6.0. TEs were counted per cell and gene using the count method from umitools v.0.5.1 with the flags --per-gene --gene-tag=XT --per-cell --wide-format-cell-counts. Clustering analysis and visualization were performed using the VarID algorithm^80^. Only cells with at least 2,000 gene transcripts or 300 transcripts derived from TEs were retained. For genes, Cells expressing >2% of *Kcnq1ot1*, a potential marker for low-quality cells^81^, were not considered. Transcripts correlating to *Kcnq1ot1* with a Pearson’s correlation coefficient >0.65, mitochondrial, ribosomal and predicted genes with Gm-identifiers were excluded. VarID was run with FSelect=TRUE, no_cores=4 and the default parameter otherwise. *t*-SNE was used for dimensionality reduction and data visualization. Differential gene and TE expression analysis was performed using the diffexpnb function of the RaceID3 algorithm similarly to a previously published method^63^. First, negative binomial distributions reflecting the gene expression variability within each subgroup were inferred on the basis of the background model for the expected transcript count variability computed by RaceID3. Using these distributions, a *P* value for the observed difference in transcript counts between the two subgroups was calculated and multiple testing corrected by the Benjamini–Hochberg method. GSEA was performed using gsePathway function of ReactomePA, an R/Bioconductor package^82^. The fold change for each gene was calculated using the diffexpnb function of VarID and was given as an argument to gsePathway function to calculate enriched gene sets using the following parameters: nPerm=1000, minGSSize=120, pvalueCutoff=0.05, pAdjustMethod=‘BH’, organism=‘mouse’.

### FLASH

pMYS-MDA5-FHBH was generated by subcloning the human MDA5 DNA sequence and the FHBH tag(3FLAG-6His-Biotin-6His) at the C-terminus of MDA5 into pMYS-IRES-GFP. HEK293T cells were transfected with pMYS-MDA5-FHBH or pMYS-GFP-FHBH with TurboFect (R0531, Thermo Fisher Scientific). The next day, cells were either irradiated (10 Gy) and collected 6 h later or treated with 1 μM decitabine (A3656, Sigma-Aldrich) for 72 h (exchanged daily). FLASH was performed as previously described^28,83^ and two replicates for each condition were sequenced using the NextSeq 500 system. The only difference with the published protocol is that washes after the streptavidin pull-down were performed with 0.1% SDS, 1 M NaCl, 0.5% LiDS, 0.5 M LiCl and 1% SDS, 0.5 M LiCl. For FLASH qPCR in HEK293 and OP9 cells, 100 µl input was taken before the first pull-down. After the streptavidin pull-down, the beads were treated with TurboDNase for 2 h for the input and 1 h followed by proteinase K (03115836001, Roche). Spike-in luciferase control RNA (15 pg μl) (L4561, Promega) was added and RNA isolation, cDNA and qPCR were performed as described above.

### FLASH analysis

FLASH-seq data were demultiplexed using Flexbar (v.3.3)^84^. For each sample, UMIs were extracted using UMITools (v.0.5.1)^85^ followed by adapter removal with TrimGalore v.0.4.4. Potential readthroughs into the barcode and UMI region were removed by clipping the last 13 bases from the 3′ ends of first mate reads. The demultiplexed and processed reads were mapped to a ribosomal consensus sequence using Bowtie2 (ref. ^67^). Reads that did not map to the ribosomal sequence were mapped to hg38 using STAR (v.2.6.0b)^26^ using the parameters ‘--winAnchorMultimapNmax 100 --outFilterMultimapNmax 100’. UMITools (v.0.5.1)^85^ was used to combine duplicated reads into individual cross-linking events. TEtranscripts (v.2.0.3)^27^ was run using both stranded options (--stranded reverse and --stranded yes). Gencode annotation (release 27) was used to define gene regions, and TETranscript was used for TE annotation. Fold change was calculated between different samples, and elements with a fold change of >1.5 and a *P* value < 0.05 (Welch approach test) were considered to be significantly bound. All .bam files were converted to bedgraphs with genomeCoverageBed a subcommand of BEDTools and were normalized using the RPKM normalization method. Graphs were generated with Prism6 (GraphPad Prism v.6.0e) (fold change cut-off of 1.5).

### Statistics and reproducibility

For cell-based experiments, biological triplicates were used in each single experiment unless otherwise stated. For animal experiments, the sample size used was determined empirically according to the nature of the experiments and is stated in the figure legends. No statistical method was used to predetermine sample size. Age-matched male and female littermates were used according to obtained genotype. For all genome-wide and FLASH experiments, 2 or 3 replicates, as stated, were used and statistical analyses were performed using the R package as stated above. A fold change cut-off of 1.5 and *P*_adj_ < 0.05 were used to determine differentially expressed genes. One transplantation experiment was excluded due to very low animal engraftment. In the single-cell RNA-seq experiments, the exclusion criteria were as follows: low quality and doublets that were filtered out computationally. High variability was observed in FLASH qPCR experiments regarding the TE copies that were bound. The experiments were not randomized. For serial CFU-C experiments, the investigators were blinded to group allocation during data collection and analysis. No other blinding was used as the nature of the experiments did not permit further blinding. Data are shown as mean ± s.d. or mean ± s.e.m. as indicated. Statistical analysis was performed using GraphPad Prism 8. Two-tailed *t*-tests were used to compare between groups and the statistical significance of the survival curve was estimated using the log-rank (Mantel–Cox) test.

### Reporting Summary

Further information on research design is available in the Nature Research Reporting Summary linked to this article.

## Online content

Any methods, additional references, Nature Research reporting summaries, source data, extended data, supplementary information, acknowledgements, peer review information; details of author contributions and competing interests; and statements of data and code availability are available at 10.1038/s41556-021-00707-9.

## Supplementary information

Reporting Summary

Supplementary Tables Supplementary Table 1. RNA-seq analysis of WT HSCs (LSK/SLAM). Differentially expressed genes H2 versus D0. Supplementary Table 2. RNA-seq analysis of WT HSCs (LSK/SLAM). Differentially expressed genes H6 versus D0. Supplementary Table 3. RNA-seq analysis of WT HSCs (LSK/SLAM). Differentially expressed genes H16 versus D0. Supplementary Table 4. RNA-seq analysis of WT HSCs (LSK/SLAM). Differentially expressed genes D3 versus D0. Supplementary Table 5. RNA-seq analysis of WT HSCs (LSK/SLAM). Differentially expressed genes D10 versus D0. Supplementary Table 6. Single−cell RNA-seq analysis of WT HSCs (LSK/SLAM). Differentially expressed genes H16 versus D0. Supplementary Table 7. ATAC-seq analysis of WT HSCs (LSK/SLAM). Gained peaks (100 kb upstream and 25 kb downstream from the TSS) H2 versus D0. Supplementary Table 8. ATAC-seq analysis of WT HSCs (LSK/SLAM). Gained peaks (100 kb upstream and 25 kb downstream from the TSS) H6 versus D0. Supplementary Table 9. ATAC-seq analysis of WT HSCs (LSK/SLAM). Gained peaks (100 kb upstream and 25 kb downstream from the TSS) H16 versus D0. Supplementary Table 10. ATAC-seq analysis of WT HSCs (LSK/SLAM). Gained peaks (100 kb upstream and 25 kb downstream from the TSS) D3 versus D0. Supplementary Table 11. ATAC-seq analysis of WT HSCs (LSK/SLAM). Gained peaks (100 kb upstream and 25 kb downstream from the TSS) D10 versus D0. Supplementary Table 12. RNA-seq analysis of WT HSCs (LSK/SLAM). Differentially expressed TEs. Supplementary Table 13. ATAC-seq analysis of WT HSCs (LSK/SLAM). Gained peaks for TEs in comparison to D0. Supplementary Table 14. Single-cell RNA-seq analysis of WT HSCs (LSK/SLAM). Differentially expressed TEs H16 versus D0. Supplementary Table 15. RNA-seq analysis of WT HSCs (LSK/SLAM). Differentially expressed TE copies. Supplementary Table 16. RNA binding to MDA5—FLASH experiment. All values. Supplementary Table 17. RNA binding to MDA5—FLASH experiment. Differentially bound TEs. Supplementary Table 18. RNA-seq analysis of *Mda5*-KO HSCs (LSK/SLAM). Differentially expressed TEs. Supplementary Table 19. Single-cell RNA-seq analysis of *Mda5*-KO HSCs (LSK/SLAM). Differentially expressed TEs H16 versus D0. Supplementary Table 20. RNA-seq analysis of *Mda5-*KO HSCs (LSK/SLAM). Differentially expressed TE copies. Supplementary Table 21. RNA-seq analysis of *Mda5-*KO HSCs (LSK/SLAM). Differentially expressed genes H2 versus D0. Supplementary Table 22. RNA-seq analysis of *Mda5-*KO HSCs (LSK/SLAM). Differentially expressed genes H16 versus D0. Supplementary Table 23. RNA-seq analysis of *Mda5-*KO HSCs (LSK/SLAM). Differentially expressed genes D3 versus D0. Supplementary Table 24. Single-cell RNA-seq analysis of *Mda5-*KO HSCs (LSK/SLAM). Differentially expressed genes H16 versus D0. Supplementary Table 25. Single-cell RNA-seq analysis of HSCs (LSK/SLAM). Differentially expressed genes D0 *Mda5-*KO versus D0 WT. Supplementary Table 26. Single-cell RNA-seq analysis of HSCs (LSK/SLAM). Differentially expressed genes H16 *Mda5-*KO versus H16 WT. Supplementary Table 27. ATAC-seq analysis of *Mda5-*KO HSCs (LSK/SLAM). Gained peaks (100 kb upstream and 25 kb downstream from the TSS) H2 versus D0. Supplementary Table 28. ATAC-seq analysis of *Mda5-*KO HSCs (LSK/SLAM). Gained peaks (100 kb upstream and 25 kb downstream from the TSS) H6 versus D0. Supplementary Table 29. ATAC-seq analysis of *Mda5-*KO HSCs (LSK/SLAM). Gained peaks (100 kb upstream and 25 kb downstream from TSS) H16 versus D0. Supplementary Table 30. ATAC-seq analysis of *Mda5* KO HSCs (LSK/SLAM). Gained peaks (100 kb upstream and 25 kb downstream from TSS) D3 versus D0. Supplementary Table 31. ATAC-seq analysis of *Mda5-*KO HSCs (LSK/SLAM). Gained peaks (100 kb upstream and 25 kb downstream from the TSS) D10 versus D0. Supplementary Table 32. RNA-seq analysis of WT myeloid cells (Mac1/Gr-1). Differentially expressed genes H16 versus D0. Supplementary Table 33. RNA-seq analysis of WT myeloid cells (Mac1/Gr-1). Differentially expressed TEs H16 versus D0. Supplementary Table 34. RNA-seq analysis of *Mda5-*KO myeloid cells (Mac1/Gr-1). Differentially expressed genes H16 versus D0. Supplementary Table 35. RNA-seq analysis of *Setdb1-*knockdown versus WT HSCs (LSK/SLAM). Differentially expressed genes. Supplementary Table 36. Oligonucleotides and others.

## Data Availability

Sequencing data supporting the findings of this study have been deposited at the Sequence Read Archive (SRA) under accession codes PRJNA532318 (FLASH data), PRJNA717283 (RNA-seq and ATAC-seq data) and PRJNA730379 (*Setdb1* RNA-seq data). Single-cell RNA-seq data have been deposited at the Gene Expression Omnibus (GEO) under accession code GSE129631. Source data are provided with this paper. All other data supporting the findings of this study are available from the corresponding author on reasonable request.

## Source data

Source Data Fig. 3 Statistical source data.
Source Data Fig. 6 Unprocessed images.
Source Data Fig. 6 Statistical source data.
Source Data Fig. 7 Statistical source data.
Source Data Fig. 8 Statistical source data.
Source Data Extended Data Fig. 2 Statistical source data.
Source Data Extended Data Fig. 3 Statistical source data.
Source Data Extended Data Fig. 3 Unprocessed images.
Source Data Extended Data Fig. 5 Statistical source data.
Source Data Extended Data Fig. 6 Statistical source data.

## Code availability

All codes used in this manuscript are available from the corresponding author on reasonable request.
