# Publisher Correction: Chemotherapy-induced transposable elements activate MDA5 to enhance haematopoietic regeneration

**DOI:** 10.1038/s41556-021-00785-9

**Published:** 2021-10-05

**Authors:** Thomas Clapes, Aikaterini Polyzou, Pia Prater, Sagar, Antonio Morales-Hernández, Mariana Galvao Ferrarini, Natalie Kehrer, Stylianos Lefkopoulos, Veronica Bergo, Barbara Hummel, Nadine Obier, Daniel Maticzka, Anne Bridgeman, Josip S. Herman, Ibrahim Ilik, Lhéanna Klaeylé, Jan Rehwinkel, Shannon McKinney-Freeman, Rolf Backofen, Asifa Akhtar, Nina Cabezas-Wallscheid, Ritwick Sawarkar, Rita Rebollo, Dominic Grün, Eirini Trompouki

**Affiliations:** 1 Department of Cellular and Molecular Immunology, Max Planck Institute of Immunobiology and Epigenetics, Freiburg, Germany; 2 Faculty of Biology, University of Freiburg, Freiburg, Germany; 3 International Max Planck Research School for Molecular and Cellular Biology (IMPRS-MCB), Freiburg, Germany; 4 Max Planck Institute of Immunobiology and Epigenetics, Freiburg, Germany; 5 Department of Medicine II, Gastroenterology, Hepatology, Endocrinology and Infectious Diseases, Freiburg University Medical Center, Faculty of Medicine, University of Freiburg, Freiburg, Germany; 6 Department of Hematology, St Jude Children’s Research Hospital, Memphis, TN USA; 7 Univ Lyon, INSA-Lyon, INRAE, BF2I, UMR0203, Villeurbanne, France; 8 Department of Computer Science, University of Freiburg, Freiburg, Germany; 9 Medical Research Council Human Immunology Unit, Medical Research Council Weatherall Institute of Molecular Medicine, Radcliffe Department of Medicine, University of Oxford, Oxford, UK; 10 Würzburg Institute of Systems Immunology, Julius-Maximilians-Universität Würzburg, Würzburg, Germany; 11 Department of Chromatin Regulation, Max Planck Institute of Immunobiology and Epigenetics, Freiburg, Germany; 12 Centre for Integrative Biological Signalling Studies (CIBSS), University of Freiburg, Freiburg, Germany; 13 Medical Research Council (MRC), University of Cambridge, Cambridge, UK

**Keywords:** Regeneration, Haematopoiesis, Haematopoietic stem cells, Quiescence

Correction to: *Nature Cell Biology* 10.1038/s41556-021-00707-9, published online 12 July 2021.

This paper was originally published under standard Springer Nature license (© The Author(s), under exclusive licence to Springer Nature America, Inc.). It is now available as an open-access paper under a Creative Commons Attribution 4.0 International license, © The Author(s). The error has been corrected in the online version of the article.

