# Supplementary material for: Chemotherapy-induced transposable elements activate MDA5 to enhance haematopoietic regeneration
Source: Nat Cell Biol. 2021 Jul 12;23(7):704–17. doi: 10.1038/s41556-021-00707-9 (PMC8492473; doi:10.1038/s41556-021-00707-9)
Supplement: Supplementary file 1 — Reporting Summary [file 41556_2021_707_MOESM1_ESM.pdf]

nature research

Corresponding author(s): Dr. Eirini Trompouki

Last updated by author(s): Jun 3, 2021

Reporting Summary

Nature Research wishes to improve the reproducibility of the work that we publish. This form provides structure for consistency and transparency in reporting. For further information on Nature Research policies, see Authors & Referees and the Editorial Policy Checklist.

Statistics

For all statistical analyses, confirm that the following items are present in the figure legend, table legend, main text, or Methods section.

n/a	Confirmed	
☐	☒	The exact sample size (*n*) for each experimental group/condition, given as a discrete number and unit of measurement
☐	☒	A statement on whether measurements were taken from distinct samples or whether the same sample was measured repeatedly
☐	☒	The statistical test(s) used AND whether they are one- or two-sided<br>*Only common tests should be described solely by name; describe more complex techniques in the Methods section.*
☒	☐	A description of all covariates tested
☒	☐	A description of any assumptions or corrections, such as tests of normality and adjustment for multiple comparisons
☒	☐	A full description of the statistical parameters including central tendency (e.g. means) or other basic estimates (e.g. regression coefficient) AND variation (e.g. standard deviation) or associated estimates of uncertainty (e.g. confidence intervals)
☐	☒	For null hypothesis testing, the test statistic (e.g. *F*, *t*, *r*) with confidence intervals, effect sizes, degrees of freedom and *P* value noted<br>*Give P values as exact values whenever suitable.*
☒	☐	For Bayesian analysis, information on the choice of priors and Markov chain Monte Carlo settings
☒	☐	For hierarchical and complex designs, identification of the appropriate level for tests and full reporting of outcomes
☒	☐	Estimates of effect sizes (e.g. Cohen's *d*, Pearson's *r*), indicating how they were calculated

Our web collection on statistics for biologists contains articles on many of the points above.

Software and code

Policy information about availability of computer code

Data collection

Sequencer: Illumina Illumina Hiseq3000, NovaSeq6000, NextSeq 500
Sequencing data demultiplexing: Illumina bcl2fastq 2.17.1
Flow cytometry: Fortessa FACS analyser, FACS ARIAIII or FACS ARIAFusion (BD Biosciences)
Imaging: LSM 880 (Zeiss), Axio Vert.A1, Zeiss
Cell counting: Casy Cell counter(OLS/Cytena).
qPCR: ABI StepOnePlus thermal cycler (Applied Biosystems), StepOnePlus Real-Time PCR machine (Applied Biosystems).

Data analysis

RNA-ATAC-FLASH

Trimmomatic (version 0.36)
STAR aligner (version -2.5.3a)
Samtools (version 0.1.19)
HTseq count (version 0.5.4p3.)
DESeq R package
gplots package (https://cran.r-project.org/package=gplots)
R package Shiny (https://shiny.rstudio.com/)
TEtranscript (Version 2.0.3)
RepeatMasker (http://www.repeatmasker.org)
Pretty Heatmaps. R package version 0.7.7. http://CRAN.R-project.org/package=pheatmap
MACS2 (version 2.1.0)
TrimGalore (Version0.4.3 and 0.4.4)
Bowtie2 (version 2.1.0)
BEDTools
DeepTools
Wellington pyDNase package (version 0.2.4)

dnase_average_profile.py of the Wellington pyDNase package
wellington_score_heatmap.py
Bowtie2 algorithm (version 2.1.0)
HOMER software59
Metascape (metascape.org)
bwa (version 0.6.2-r126)
umitools 0.5.1
featureCounts from the subread-1.5.3 package
Samtools 1.6.0.
VarID algorithm
RaceID3 algorithm
GSEA was performed using gsePathway function of ReactomePA, an R/Bioconductor package
Flexbar (version 3.3)
UMITools (Version 0.5.1)

Flow cytometry: FlowJo 10.6.1.
Imaging: Imaris software 9.2

For manuscripts utilizing custom algorithms or software that are central to the research but not yet described in published literature, software must be made available to editors/reviewers. We strongly encourage code deposition in a community repository (e.g. GitHub). See the Nature Research guidelines for submitting code & software for further information.

Data

Policy information about availability of data

All manuscripts must include a data availability statement. This statement should provide the following information, where applicable:

- Accession codes, unique identifiers, or web links for publicly available datasets
- A list of figures that have associated raw data
- A description of any restrictions on data availability

DATA AVAILABILITY
Sequencing data that support the findings of this study have been deposited in the Short Read Archive SRA under accession codes PRJNA532318 (FLASH data), PRJNA717283 (RNA and ATAC-seq data) and PRJNA730379 (SETDB1 RNA-seq data). Single cell RNA-sequencing data have been deposited in the gene expression omnibus (GEO) under accession code GSE129631. All other data supporting the findings of this study are available from the corresponding author on reasonable request.

CODE AVAILABILITY
All codes used in this manuscript are available from the corresponding author upon reasonable request.

Field-specific reporting

Please select the one below that is the best fit for your research. If you are not sure, read the appropriate sections before making your selection.

☒ Life sciences ☐ Behavioural & social sciences ☐ Ecological, evolutionary & environmental sciences

For a reference copy of the document with all sections, see nature.com/documents/nr-reporting-summary-flat.pdf

Life sciences study design

All studies must disclose on these points even when the disclosure is negative.

Sample size	The sample size was determined empirically according to the nature of the experiments. Animal experiments had increased sample size in comparison to cell based experiments.
Data exclusions	One transplantation experiment was excluded due to very low animal engraftment. In the single-cell RNA-seq experiments the exclusion criteria were: low quality and doublets that were filtered out computationally
Replication	The number of replicates and independent experiments is indicated in the figure legends. Besides the one transplantation experiment replication attempts were successful. High variability was observed in FLASH qPCR experiments regarding the TE copies that were bound.
Randomization	The experiments were not randomized. Age-matched male and female littermates were used according to obtained genotype.
Blinding	For serial CFU-C experiments the investigators were blinded to group allocation during data collection and analysis. No other blinding was used since the nature of the experiments did not permit further blinding.

Reporting for specific materials, systems and methods

We require information from authors about some types of materials, experimental systems and methods used in many studies. Here, indicate whether each material, system or method listed is relevant to your study. If you are not sure if a list item applies to your research, read the appropriate section before selecting a response.

Materials & experimental systems

n/a	Involved in the study
☐	☒ Antibodies
☒	☐ Eukaryotic cell lines
☒	☐ Palaeontology
☐	☒ Animals and other organisms
☒	☐ Human research participants
☒	☐ Clinical data

Methods

n/a	Involved in the study
☒	☐ ChIP-seq
☐	☒ Flow cytometry
☒	☐ MRI-based neuroimaging

Antibodies

Antibodies used

All antibodies were purchased from Biolegend and used in 1:400 dilution unless indicated otherwise. CD45.1/Ly5.1 (APC-Cy7, clone A20), CD45.2/Ly5.2 (Alexa Fluor 700, clone 104), CD3e (FITC, clone 145-2C11), CD11b/Mac-1 (1:1600, FITC or PerCP-Cy5.5, clone M1/70), Ly6C/Ly6G (1:1600, FITC or PerCP-Cy5.5, RB6-8C5), CD45R/B220 (FITC or APC, Clone RA3-6B2), Ter119 (FITC, clone Ter-119), CD117/c-kit (Brilliant Violet 421[1:600] or PE; Biolegend, or APC-H7, clone 2B8, [1:200] BD Bioscience,), Sca-1 (Pe-Cy7, clone E13-161.7), CD48 (1:800, PerCP-Cy5.5, clone HM48-1), CD150 (1:600, PE-Dazzle or 1:600 Brilliant Violet 605, Clone TC15-12F12.2), CD135/Flk2 (1:200, PE, clone A2F10.1, BD Pharmingen), CD34 (1:30, Alexa Fluor 700 clone RAM34, eBioscience), Ki67 (1:200, Alexa Fluor 647, clone 11F6), CD201 (1:200, EPCR, PE anti-mouse clone RCR16), p-IRF3 (1:25, S396, clone D601M, Rabbit mAb 29047, Cell Signaling), goat anti rabbit secondary antibodies (1:500, Alexa Fluor 647, A21245, Invitrogen), γH2AX antibody (1:100, Alexa Fluor 647 (Ser139), clone 2F3), p65 (1:100, Alexa Fluor 488, p65, Santa Cruz Biotechnologies). For the LINE1 knockdown experiments: Sca-1 (1:200, PerCPCy5.5 122523, E13-161.7clone, Biolegend), c-Kit (1:200, APCe780, 47-1171-82, clone 2B8, eBioscience), CD48-Alexa Fluor700 (1:200, 56-0481-82, clone HM48-1, eBioscience) and CD150 (1:200, PE-Cy7, 115913, clone TC15-12F12.2, Biolegend).

Validation

Validation by manufacturer.APC-Cy7 anti-CD45.1/Ly5.1, Alexa Fluor 700 anti-CD45.2/Ly5.2, FITC anti-CD3e, APC or FITC anti-CD45R/B220, Pe-Cy7 anti-Sca-1, PerCP-Cy5.5 or AF-700 anti-CD48, BV-605 or PE-Cy7 or PE-Dazzle anti-CD150, PerCPCy5.5 anti Sca1, APCe780 anti c-kit, CD48-Alexa Fluor700, AF-647 anti-Ki67, – flow cytometry on mouse splenocytes
FITC or PerCP-Cy5.5 anti-CD11b/Mac-1, FITC or PerCP-Cy5.5 anti-Ly6C/Ly6G, FITC anti-Ter119, BV-421 or PE or APC-H7 or APCe780 anti-CD117/c-kit, AF-700 anti-CD34, – flow cytometry on mouse bone marrow cells
PE anti-CD135/Flk2 – flow cytometry on mouse bone marrow leukocytes
PE anti-CD201/EPCR – flow cytometry on HUVEC cells
AF-647 anti-γH2AX – imaging on Nocodazole-treated Hela cells

Animals and other organisms

Policy information about studies involving animals; ARRIVE guidelines recommended for reporting animal research

Laboratory animals

Mda5-/- mice (B6.Cg-Ifih1tm1.1Cln/J)44 were purchased from the Jackson Laboratory and backcrossed 10 times into C57BL/6J WT mice (CD45.2+/Ly5.2). Sting-/- mice bones (B6(Cg)-Sting1tm1.2Camb/J)55, and Mavs-/- mice bones (Mavstm1Tsc)56 and their respective controls, were a kind gift from Jan Rehwinkel. All animals were were maintained in the animal facility of the Max Planck Institute of Immunobiology and Epigenetics under specific pathogen free conditions in individually ventilated cages with a light-dark cycle of 12 hours at 20-24oC with 45-65% humidity. For all genotypes, 6- to 12-week-old age and gender-matched female or male mice were used in the experiments.

Wild animals

This study did not involved wild animals

Field-collected samples

This study did not involved field-collected samples.

Ethics oversight

All mouse experiments were carried out in accordance to the guidelines of the Federation of European Laboratory Animal Science Association and following legal approval of the Regierungspräsidium Freiburg (35/9185.81/G-15/100, 35-9185.81/G-18/41, 35-9185.81/G-18/127, 35-9185.81/G-20/127).

Note that full information on the approval of the study protocol must also be provided in the manuscript.

Flow Cytometry

Plots

Confirm that:

☒ The axis labels state the marker and fluorochrome used (e.g. CD4-FITC).

☒ The axis scales are clearly visible. Include numbers along axes only for bottom left plot of group (a 'group' is an analysis of identical markers).

☒ All plots are contour plots with outliers or pseudocolor plots.

☒ A numerical value for number of cells or percentage (with statistics) is provided.

Methodology

Sample preparation	Bone marrow from tibiae, femurs and hip bones were crushed in staining buffer (PBS supplemented with 2 %FBS and 1 mM EDTA) using a mortar and pestle and filtered through a 70-µm cell strainer (Flacon, 352350) to isolate the BM cells. Red cells were lysed in an Ammonium-Chloride-Potassium Buffer (ACK-Lysis Buffer, NH4Cl 150 mM, KHCO3 10 mM, EDTA 0.1 mM) for 5 min at room temperature. Cells were then washed with staining buffer.<br>For the LINE1 knockdown experiment BM was extracted from femur, pelvic bone, tibias and spine via crushing. Further preparation is described in the Methods section.
Instrument	Data were either acquired on a Fortessa FACS analyser (Becton Dickinson) or sorted using a FACS ARIA III or FACS ARIA Fusion (Becton Dickinson).
Software	FlowJo 10.6.1
Cell population abundance	Purity check was perfomed on a more abundant population (LSK CD48+CD150+) as the number of HSCs after sorting was on average 3,000-5,000 per animals.
Gating strategy	Unstained population and single staining controls were used to to both gating strategy and correct spectral overlaps. The gating strategy can be found in Extended Data Fig. 1 and the markers used for each population can be found in the section "Sorting strategy" in the methods section.

☒ Tick this box to confirm that a figure exemplifying the gating strategy is provided in the Supplementary Information.
