# Supplementary material for: Chemotherapy-induced transposable elements activate MDA5 to enhance haematopoietic regeneration
Source: Nat Cell Biol. 2021 Jul 12;23(7):704–17. doi: 10.1038/s41556-021-00707-9 (PMC8492473; doi:10.1038/s41556-021-00707-9)

Unprocessed images Fig. 6d

WT_D0_Hoechst

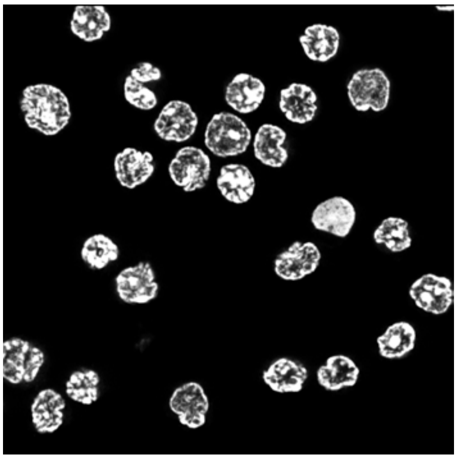

WT_D0_p65

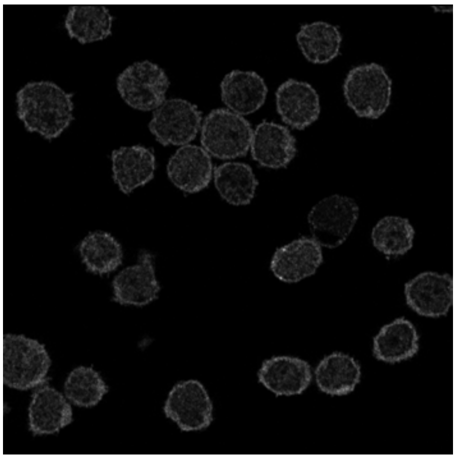

*MDA5-/-*_D0_Hoechst

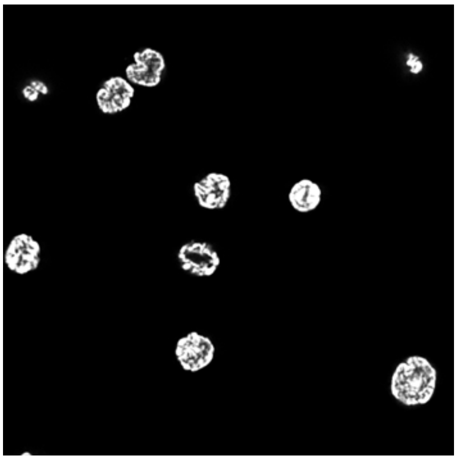

*MDA5-/-*_D0_p65

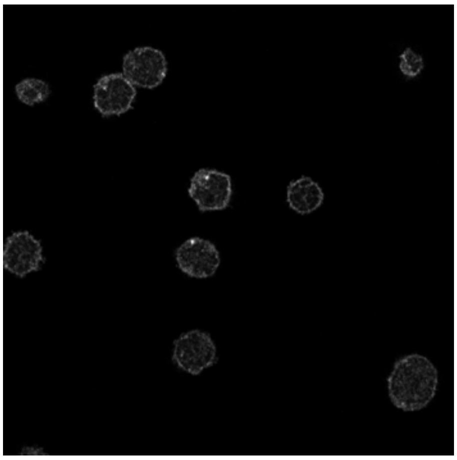

WT_H16_Hoechst

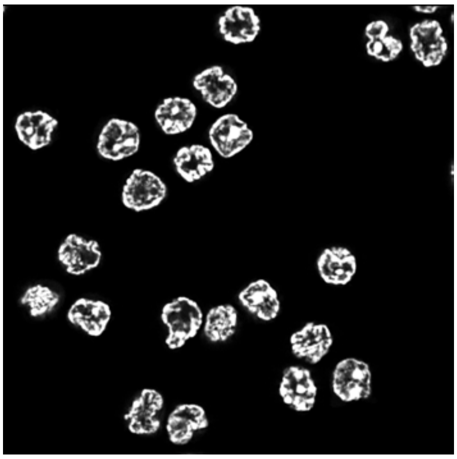

WT_H16_p65

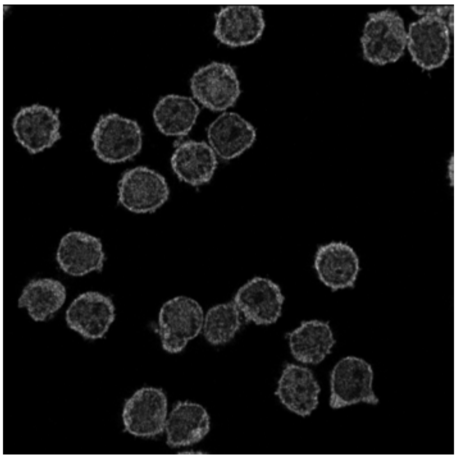

*MDA5-/-*_H16_Hoechst

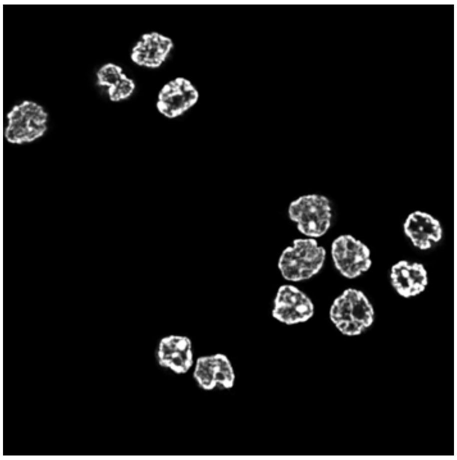

*MDA5-/-*_H16_p65

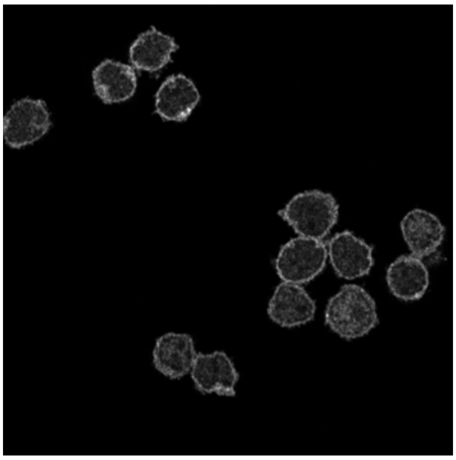

Supplement: Source Data Fig. 6 — Unprocessed images. [file 41556_2021_707_MOESM4_ESM.pdf]
