# Supplementary figures and images for: Chemotherapy-induced transposable elements activate MDA5 to enhance haematopoietic regeneration
Source: Nat Cell Biol. 2021 Jul 12;23(7):704–17. doi: 10.1038/s41556-021-00707-9 (PMC8492473; doi:10.1038/s41556-021-00707-9)

WT

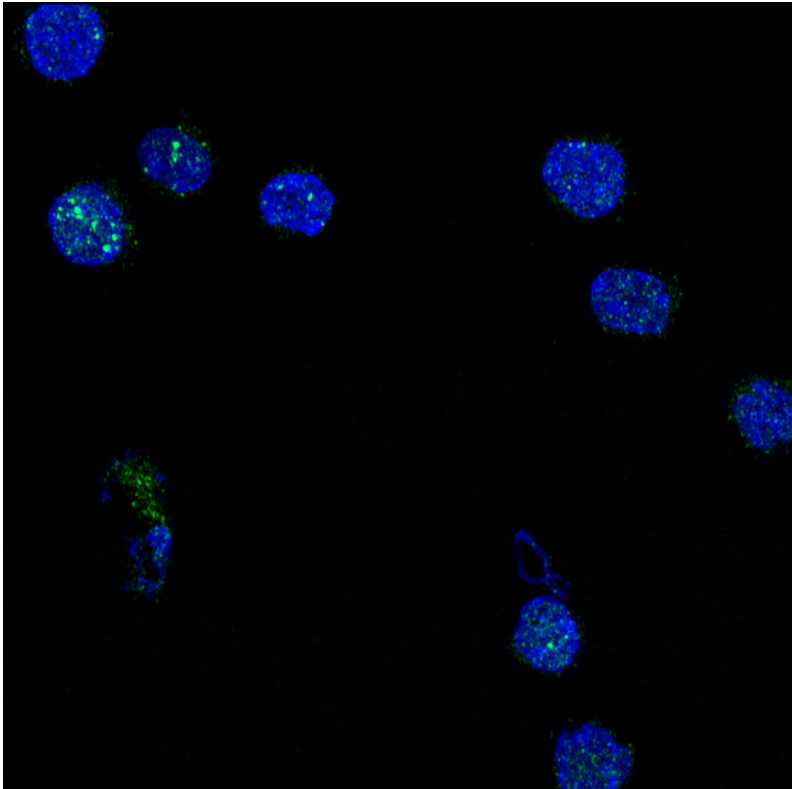

*MDA5*<sup>-/-</sup>

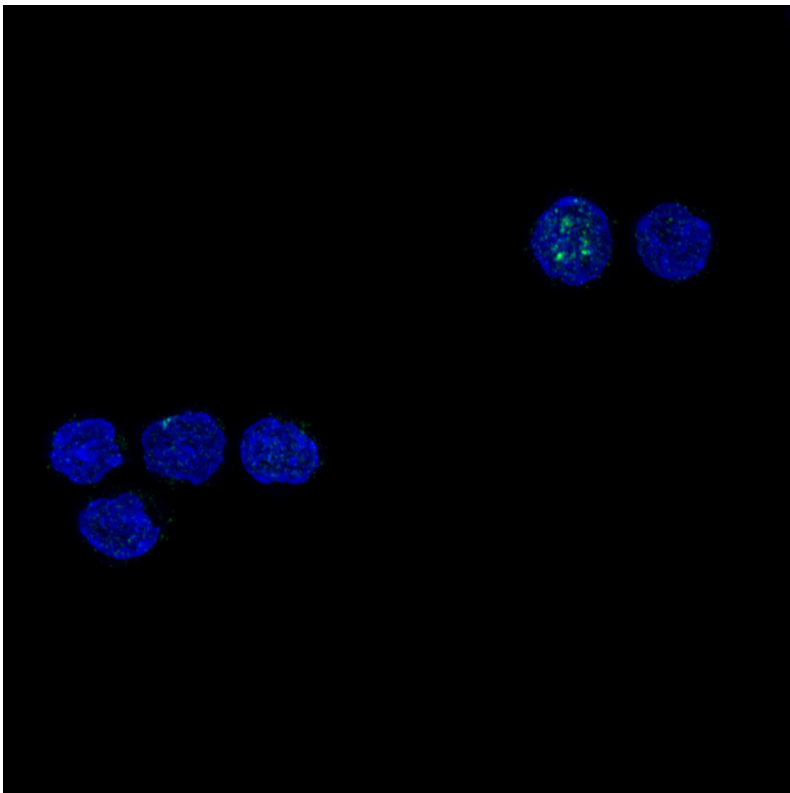

Supplement: Source Data Extended Data Fig. 3 — Unprocessed images. [file 41556_2021_707_MOESM10_ESM.pdf]
